# A Generic Requirement Engineering Framework for Social Commerce Platforms: Front-end and Back-end Features

**DOI:** 10.12688/f1000research.170127.1

**Published:** 2025-10-09

**Authors:** Iman Al Jassasi, Youcef Baghdadi, Abdullah Al Hamdani

**Affiliations:** 1 Computer Science, Sultan Qaboos University, Al Khoudh, Muscat, 123, Oman

**Keywords:** e-commerce; s-commerce, framework, social features, design model, requirement engineering, front-end requirements, back-end requirements.

## Abstract

**Background:**

Social commerce (s-commerce) represents the convergence of social media and electronic commerce, where online shopping is integrated with social networking functionalities enabled by Web 2.0 technologies. As a driver of digital transformation, s-commerce continues to evolve rapidly in response to technological advancements and shifting consumer behaviors. Despite its growing, research addressing the systematic development of platforms that support this business model, particularly in relation to the requirements engineering (RE) process remains limited.

**Method:**

This study applies the Design Science Research Methodology (DSRM) to design and evaluate a comprehensive RE framework for s-commerce platforms. The six DSRM steps were followed: problem identification, objectives of a solution, design and development, evaluation, and communication. The framework integrates variability concept through requirements reuse. A structured knowledge base of front-end requirements, formalized with Feature Description Language (FDL) and Unified Modeling Language (UML), ensures consistency, traceability, and reusability. Evaluation was conducted through prototype artifacts developed using Visual Studio Code, Docker Desktop, and TableTools, as well as through meta-criteria assessment. Moreover, the framework was evaluated through expert’s evaluation Central ofInformation System (CIS)developers from Sultan Qaboos University (SQU). The steps of the method were implemented and tested over a period from January 2023 to September 2025.

**Results:**

The framework is implemented as a web-based prototype artifact that establishes a structured process for requirements elicitation, specification, negotiation, validation, and management. It consolidates a generic set of 28 front-end and 15 back-end requirements for s-commerce platforms, demonstrating their practical use in deriving platform-specific requirements. The framework’s prototype is supported by two complementary tools: generates requirements and recommends additional requirements in the knowledge base.

**Conclusion:**

This study provides actionable guidance for e-commerce and s-commerce developers. By focusing on requirements that enhance economic value, strengthen customer relationships, and improve platform architecture, the framework contributes to more reliable, adaptable, and efficient s-commerce systems.

## 1. Introduction

The global popularity of social media has grown substantially in recent years, with adoption rates rising at an unprecedented pace. ^1^ Over the last four decades, technological developments have profoundly reshaped society. ^2^ Globalization has removed trade barriers and promoted a knowledge-based economy, with technology transforming numerous aspects of human life particularly social interactions. ^2^ Within this context, social commerce (s-commerce) has emerged as a paradigm that leverages social media platforms and networks to facilitate the online buying and selling of products and services. ^3, 4^ S-commerce has also been defined as the adoption of social interaction features that strengthen consumer–vendor relationships. ^5^ By integrating social interactions with electronic commerce (e-commerce), s-commerce enables users to discover, share, and purchase items directly within social media environments. ^6^ It plays a pivotal role in the digital transformation of society by empowering participation through social interactions and community engagement. The rise of Web 2.0 technologies has accelerated this transformation, fostering the integration of online commerce platforms as an alternative to traditional retail experiences. ^3, 6, 7^

From a business perspective, s-commerce has attracted substantial academic attention, with research exploring its economic, behavioral, and managerial implications. ^8, 9^ From an information technology (IT) perspective, however, the development of s-commerce platforms remains a formidable challenge. Designing platforms that effectively support s-commerce business models requires establishing a minimum common set of social requirements and features. ^5, 10^ These requirements are considerably more complex than those of traditional e-commerce, ^11^ as they span both front-end and back-end dimensions. Front-end requirements capture interactions with participants, communities, and social features, while back-end requirements address how user-generated content can be leveraged to improve product and service architectures and enhance business processes. ^3^ The diversity and complexity of these requirements make systematic development processes essential, particularly robust requirements engineering (RE) practices.

RE encompasses the elicitation, specification, negotiation, validation, and management of software requirements. ^12, 13^ It further emphasizes the distinction between commonalities and variabilities across systems. ^14^ In the context of s-commerce platforms, rigorous RE practices minimize the gap between developers and users. ^15^ Without such practices, projects risk failure, resulting in brittle platforms that fail to identify critical user groups, their communities, interactions, and the content generated from these interactions.

S-commerce presents numerous opportunities for interaction among consumers, companies, and public organizations, creating mutually beneficial products and experiences. Yet, debates persist regarding the underlying motivations of consumer behavior and the methods necessary to establish s-commerce as a sustainable business model. ^1^ At the same time, risks such as privacy concerns, misinformation, and the complexity of requirement specification continue to challenge its adoption. ^2^ While prior studies have examined s-commerce primarily from a design or empirical perspective, very few have addressed its development from RE standpoint. This gap underscores the need for systematic frameworks that can guide the RE process in s-commerce platform development.

To address this gap, this study proposes a framework that consolidates the commonalities of s-commerce platforms into a generic and complete set of requirements covering both front-end and back-end dimensions. The objectives of this study are to:

• Assist developers in generating platform requirements from a knowledge base of generic requirements.
• Support negotiation, prioritization, and the addition of new requirements to the knowledge base.
• Provide an instantiation mechanism that employs variability techniques to recommend specific subsets of requirements tailored to particular platforms.
• Enable the specification of requirements using Standard Natural Language (SNL), Unified Modeling Language (UML) use case diagrams, or alternative modeling techniques for validation.
• Facilitate systematic requirements management throughout the platform’s lifecycle.
• Enhance existing s-commerce development models by embedding a systematic RE process, thereby addressing a common cause of project failure.

The framework was developed using the six-step Design Science Research Methodology (DSRM). ^16^ This paper focuses on the first three steps: defining the problem and justifying the need for the framework, specifying the objectives of the solution, and outlining the design of the framework. These steps were supported by a literature review, two complementary research methods a systematic literature review (SLR) and a systematic mapping study (SMS). The framework adopts an artifact-oriented paradigm, as its purpose is to support developers in generating specific subsets of requirements rather than prescribing detailed RE activities.

This study advances both theoretical and practical understanding of s-commerce. Academically, it enriches the literature by bridging the gap between s-commerce design and RE. Practically, it provides an instantiated framework that developers can apply to strengthen the competitiveness of s-commerce platforms by systematically addressing requirements across front-end and back-end dimensions.

The paper includes a structured abstract, an introduction outlining the research problem and objectives, and a literature review highlighting gaps in existing s-commerce frameworks. The methodology section explains the DSRM process and prototype development, while the results section presents front-end and back-end requirements, variability management, and tool features. The evaluation section presents the three methods adopted in this study: prototype-based evaluation, meta-criteria assessment, and expert evaluation, followed by a discussion comparing the framework with prior work. The paper concludes with a conclusion section including contributions, limitations, and future work, along with standard statements.

## 2. Literature review

### 2.1 Transferring e-commerce enterprises to s-commerce

Based on, ^9^ the most general definition identifies s-commerce, or s-commerce, as the use of social media or networks to facilitate user involvement in selling products, sharing information, and shopping online. However, s-commerce differs in many aspects from e-commerce. Recently, enterprises have been paying attention to how to conduct commercial activities on social networking sites ^3, 6^; accordingly, many researchers believe the following: First, s-commerce platforms intend to influence society and persuade prospective customers to make a purchase decision by establishing a direct interaction with them. ^5, 17^ Second, the development of s-commerce allows a business to implement two ways of interaction compared to e-commerce, where the interactions are conducted only in one way. ^3^ Third, social features on an s-commerce website provide value for both the consumer and the enterprise, as the consumer can be approached in commercial activities by other buyers and decisions are influenced by the community. The enterprise will improve as it gains recommendations from the consumer and some positive recommendations will attract new consumers. ^18^ Fourth, s-commerce extends the opportunities to develop customer relationships; it also enhances web traffic, unleashes better business prospects, and improves brand identity. ^4^ Fifth, the COVID-19 pandemic has sped up the adoption of technology and increased the move towards online interactions, such as s-commerce. ^2^ Table 1 shows the aspects to focus on when deciding whether to transfer an e-commerce platform to an s-commerce platform.

**Table 1.** Highlighted aspects between e-commerce and s-commerce.

Aspect	E-commerce	S-commerce
Business Model	• The architecture of the products or services and the modeling and design of the business processes are limited to the enterprise and/or its partners.	• Many actors participate in the elaboration of the architecture of the products/services and the modeling and design of the business processes.
Interaction	• The customer interacts individually with the e-commerce websites and independently from other customers. • It deals with customers as individuals.	• Online communities support social connections to enhance conversations between customers.
Design	• Content, structure, and navigation. • Content: the characteristics of the products/services, the shopping cart, and, to a lesser extent, the customer profiles. • Structure: designed around the products/services and the shopping cart. • Navigation: the use of discovery mechanisms such as links, searching, and browsing the shopping cart.	• Individual, interactions, community, and generated content. • Content, structure, and navigation: user-centered design, through an interactive interface that enables identity, interactions, and communities as well as user-created/shared content. • Social features: like, share, rate, review, and recommend.

### 2.2 The current s-commerce requirements frameworks

Most existing research on s-commerce requirements has concentrated on the business perspective or examined the phenomenon in terms of capabilities, adoption factors, user perceptions, and the role of social networking platforms. For instance, ^19^ introduced the concept of social electronic business (e-business), presenting a vision that merged web-based collaborative tools with the advantages of social networking and social capital. Similarly, ^19^ integrated Web 2.0 functionalities such as RSS feeds, forums, and wikis into the collaborative development of an ERP system (the OpenBravo project) for SMEs, thereby enhancing business collaboration and introducing a social dimension to enterprise systems.

Several studies have proposed frameworks to analyze the drivers of s-commerce adoption. For example, ^20^ examined technological, organizational, and environmental factors influencing SME adoption, demonstrating positive impacts on productivity and income. Likewise, ^21^ highlighted how social network analysis fosters knowledge sharing, creativity, trust-building, and cognitive capital, but also identified challenges including information overload, lack of standardization, privacy risks, time constraints, limited access, skill requirements, and financial costs.

Other research has focused on structuring the design of s-commerce platforms. In, ^22^ a framework was introduced to describe the technological and conceptual elements of s-commerce, their interrelationships, and constraints, outlining a six-step process for platform design. Building on this, ^23, 24^ proposed a four-element framework (customer, merchant, platform, and context) to guide both design and evaluation, supported by metrics to assess platform potential. Similarly, ^25^ examined how s-commerce design influences consumer decision-making, emphasizing the role of social factors in shaping purchasing behavior.

From a technical perspective, ^26^ emphasized that both functional (F) and non-functional (NF) requirements are critical to the effectiveness of s-commerce platforms yet remain insufficiently explained in existing studies. Their proposed design model sought to capture these technical attributes more explicitly. Likewise, ^27^ introduced the Social Presence Model (SPM), which identifies social requirements such as feedback, communication, ratings, recommendations, reviews, rankings, comments, and advertisements that help bridge e-commerce and s-commerce. In parallel, social support theory reinforces the importance of recommendations and referrals in enhancing user engagement. ^28^ Finally, ^10, 29^ highlighted four core functional requirements of s-commerce: conversation, community, commerce, and individuals. Their work focused on the promotional features of platforms, showing how these elements can maximize online sales and strengthen consumer engagement.

Despite these valuable contributions, current frameworks are fragmented and remain largely business- or feature-oriented. They emphasize adoption factors, social interactions, or promotional features, but overlook the need for a systematic RE process. In particular, existing studies rarely distinguish between front-end requirements (user interactions, communities, and social features) and back-end requirements (leveraging user-generated content to improve products, services, and business processes). Without an integrated RE approach, the complexity and variability of s-commerce platforms cannot be effectively addressed. This gap underlines the necessity for a comprehensive framework that consolidates common requirements, manages variability, and supports developers in systematically engineering robust s-commerce platforms. 

## 3. Methods

This study adopts the DSRM as the overarching approach to design, develop, and evaluate the proposed RE framework for s-commerce platforms. The DSRM consists of six iterative steps, each producing specific outputs that contribute to the construction and validation of the research artifact.

### Step 1: Problem identification

We identify the research problem; two reviews were undertaken: an SMS and a SLR. The SMS explored the question: “What are the existing design models or frameworks of s-commerce platforms?” Its rationale was to analyze current models and design processes and to determine whether existing approaches adequately assist developers. Results showed that most prior studies investigated s-commerce from a design perspective only, offering little guidance on the overall development process.

The SLR addressed the question: “What are the social features (requirements) of s-commerce platforms?” Its rationale was to identify the social requirements necessary to extend e-commerce into s-commerce. The review consolidated features such as participation, content sharing, community interaction, and recommendation functions, which represent critical front-end requirements.

Together, the SMS and SLR revealed significant gaps: existing frameworks lack systematic RE processes, guidelines for integrating both front-end and back-end requirements are missing, and the transformation from e-commerce to s-commerce remains unclear. Without a rigorous RE framework, platforms risk being fragile, poorly adaptable, and unable to meet user needs.

### Step 2: Objective of a solution

The objective of the solution is provided as a generic framework. This framework will be used by developers to generate a set of sound requirements of their specific s-commerce platforms from two perspectives: front-end and back-end.

### Step 3: Framework design and development

The design and development stage transformed the conceptual objectives into a tangible artifact through four main activities:

**Requirements collection and analysis:** Conducted via a SLR, feature extraction from real-world s-commerce platforms, and integration of insights from existing RE approaches. This yielded a comprehensive set of requirements covering front-end (participants, community, content, social features) and back-end (service architecture, security, transaction management) perspectives.

**Knowledge base construction:** The requirements were formalized into a knowledge base of 28 front-end and 15 back-end requirements. Feature Description Language (FDL) was used for textual formalization, while UML diagrams visually represented requirement relationships and scenarios. This dual documentation ensured clarity, traceability, and extensibility.

### 3.1 Variability management and instantiation mechanism

The variability of the s-commerce platforms, as not all the platforms are similar, (i.e., different markets have different platforms and different requirements set). The framework incorporates variability concept to ensure adaptability across diverse s-commerce platforms. Specifically, it applies **requirement reuse** to leverage previously defined knowledge, targeting three types of dimensions: (Requirements type selection, Domain type selection and, Social Feature type selection). These enable the transformation of a generic set of requirements into platform-specific configurations while systematically documenting dependencies, constraints, and reuse rules. By doing so, the framework not only supports efficient tailoring but also minimizes bias, redundancy, and inconsistencies in the RE.

### 3.2 Tool development

A web-based tool was developed to operationalize the framework. It integrates the knowledge base and supports developers in requirement elicitation, specification, negotiation, validation, and management. It also automates the instantiation process, generating platform-specific requirement sets and prototype specifications.

### 3.3 Development environment and technologies

The prototype development and code management were facilitated through the combined use of Visual Studio Code, which served as the primary integrated development environment (IDE) for coding and debugging; Docker Desktop, which enabled containerization and ensured a consistent execution environment across different deployment settings; and TablePlus, which was employed for efficient database management, query execution, and schema organization. The primary implementation language adopted for the framework was.NET, chosen for its robustness, scalability, and compatibility with enterprise-level web applications. Together, these tools provided a stable, flexible, and reproducible development environment that supported both the implementation of the prototype and the maintenance of its underlying codebase.

### Step 4: Demonstration

The framework and tool were applied in a case study with a small–medium enterprise in Oman. Developers used the tool to generate platform-specific requirements and instantiate prototype specifications. Feedback was discussed with stakeholders within the Central Information System (CIS) at Sultan Qaboos University, focusing on selected components of the student portal, in order to further evaluate its applicability and effectiveness in an academic context.

### Step 5: Evaluation

The framework was evaluated using a multi-pronged strategy:

• Prototype-based testing confirmed the ability of the tool to generate platform-specific requirements and manage them across the lifecycle.
• Comparative analysis against existing frameworks was conducted using the meta-criteria and the clustering-aided assessment of Kraiem which was developed in 2022.
• Expert review by domain specialists assessed the framework’s consistency, traceability, reusability, and practical utility.
• Bias control measures included triangulation of data sources (literature, case study, expert input), independent reviews, and cross-validation of results.

### Step 6: Communication

The final step of the DSRM involved the dissemination of findings. Results were communicated through scientific publication and engagement with industry stakeholders. The enterprise partner was introduced to the framework and tool to promote adoption, practical refinement, and sustainability.

### 3.4 Timeframe of each step of DSRM

Table 2 outlines the timeframe associated with each phase of the DSRM as implemented in this research.

**Table 2.** Timeframe of each step of DSRM.

DSRM Step	Timeframe
Problem Identification	January – March 2023
Define the Objectives of a Solution	April – June 2023
Design and Build in & Development	July 2023 – June 2024
Demonstration	July – December 2024
Evaluation	January – June 2025
Communication	July – September 2025

## 4. Results based on DSRM

### 4.1 Problem identification

This study employed two complementary literature reviews to identify the research problem. First, an SMS was conducted to examine existing design models of s-commerce platforms. Second, a SLR was performed to consolidate the social features of s-commerce platforms into a set of generic requirements.

• **An SMS**

This section is structured to address the following research question and its corresponding findings:

*SMSQ1: What are the existing design models of s-commerce platforms?*

To answer SMSQ1, we conducted an in-depth analysis of the existing models and design processes proposed for s-commerce platforms. The objective was to examine whether any systematic design approaches currently exist to guide developers in building such platforms. Our review revealed several models, which are summarized as follows:

**First: Information-Model (I-Model)**

According to, ^30^ the I-model encompasses four key entities: information, people, technology, and management. The information layer illustrates the transition from e-commerce to s-commerce, the technology layer is associated with social networks and media-sharing platforms, the management (or organizational) layer incorporates strategies such as the long-tail approach, and the people layer reflects human social attributes. Similarly, ^31^ emphasized the same four foundational elements of s-commerce business, technology, people, and information suggesting conceptual alignment between the two studies. It can therefore be observed that both ^30, 31^ converge on essentially the same design model for s-commerce. Extending this line of inquiry, ^32^ analyzed the I-model from broader perspectives by incorporating the four original components within a platform layer while also introducing additional layers, namely customers, merchants, and contextual factors.

While these studies provide valuable insights into the structural foundations of s-commerce, their focus remains largely descriptive and high-level. They lack explicit guidance on how such layered elements can be systematically operationalized during RE and development processes. This limitation highlights the need for a more comprehensive framework, one that not only identifies the essential dimensions of s-commerce but also integrates them into a practical, variability-driven approach capable of supporting customization, traceability, and scalability across diverse platform contexts.

**Second: The five-layer model**

The design model of s-commerce proposed in ^10^ comprises four primary layers: individual, conversation, community, and commerce. To advance this structure, the authors of ^30^ extended the model by introducing an additional management layer. The purpose of each layer can be summarized as follows: the individual layer relates to user profiles and participant identification; the conversation layer facilitates communication among users through social tools that enable self-representation, information sharing, and content generation; the community layer focuses on sustaining customer relationships by offering social and technical support; and the commerce layer enables core business activities such as buying and selling. Finally, the management layer was introduced to capture, organize, and leverage information and customer experiences after commercial transactions, thereby supporting long-tail strategies.

**Models and frameworks analysis**

An SMS indicated that most of the selected works pursued one or more of the following objectives:

• Providing support for developers during the design phase of platform development.
• Proposing social features to be embedded in s-commerce platforms.
• Enhancing customer satisfaction by considering user preferences toward social features.
• Focusing on social mechanisms that attract customer attention and increase enterprise revenues.

Despite these contributions, the review revealed notable limitations. Most proposed models and frameworks remain primarily structural and descriptive, offering neither explicit guidance for front-end requirements (e.g., user interface, interaction design, usability) nor back-end requirements (e.g., scalability, security, integration). Instead, they largely emphasize platform layers and social features without addressing the underlying RE processes. Critically, the requirements analysis phase, a cornerstone of any robust system design, was entirely absent. This omission represents a significant gap, as neglecting requirements analysis undermines the effectiveness of the design phase and, ultimately, the successful development of s-commerce platforms.

• **The SLR**

To establish a sound set of generic requirements, SLR was conducted to examine the social features of s-commerce that enhance the functionality of such platforms. This review was also intended to critically validate the necessity of a structured guideline to support developers in designing and implementing s-commerce platforms. The primary objective of the review was to define and consolidate the social features discussed in previous studies, thereby providing a foundation for our proposed framework.

In the SLR, we addressed the following research question:

*SLRQ2: What are the social features of s-commerce platforms?*

By answering this question, we aimed to identify the set of social requirements essential for transforming a conventional e-commerce platform into an s-commerce platform. Based on the thematic areas of the selected studies, the social features of s-commerce can be broadly categorized into three dimensions: (i) features based on building blocks, (ii) features based on the four-layer model, and (iii) features based on social principles.

i. **Social features based on S-Commerce building blocks**

Compared to e-commerce, s-commerce platforms incorporate more complex building blocks and constructs. These include participants, communities, user-generated content, and enterprise social interactions. ^3, 33^ According to, ^3, 22^ the building blocks of s-commerce are organized into four layers: participants, community, content, and social interactions.

• The participants layer comprises the actors interacting within the platform, such as customers, employees, partners, and suppliers.
• The community layer represents collaborations among multiple participants, enabling collective decision-making and support.
• The content layer includes any multimedia or textual material contributed by actors.
• The social interactions layer links all of these components, requiring at least two elements to interact simultaneously.

The enterprise’s social interaction layer is particularly critical, as it provides the foundation for creating a social environment within the platform. ^3, 22^ It enables participants to interact using well-established mechanisms such as SLATES (Search, Link, Author, Tag, Extend, Signal) and 4RC (Rank, Rate, Recommend, Review, Comment) features. ^3, 34^

However, integrating such enterprise social interactions into existing e-commerce systems is a non-trivial challenge. It requires careful consideration of multiple factors, including security, privacy, participant support, content management, performance, and scalability. ^22^ Consequently, developing effective s-commerce platforms involves addressing not only functional design requirements but also broader issues related to business modeling, relationship management, and compliance with privacy expectations.

To support enterprise-level social interactions, there is also a need for robust technological architecture, particularly Web 2.0 services, cloud computing, and service-oriented architectures (SOA). These technologies act as the enablers of social interaction features and form the backbone of platform integration.

Table 3 presents a summary of the social features associated with s-commerce building blocks, as discussed in. ^3, 10, 30, 35– 37^ Furthermore, ^26^ explored both functional and non-functional features that s-commerce platforms require. Functional features were classified into five distinct categories as shown in Table 4, while non-functional features included information quality, system quality, service quality, and usability.

**Table 3.** Social features of s-commerce building block. ^1, 8, 28, 31– 33^

Element	Features
**Participants**	•Eligibility for registration and login. •Ability to engage in social features such as 4RC (rank, rate, recommend, review, comment) and SLATES (search, link, author, tag, extend, signal). •Support for direct communication tools, including chat and call options.
**Community**	•Real-time support from the platform team. •Peer-to-peer support from other community members. •Facilities for exchanging ideas and mutual interaction. •Provision of emotional, informational, and technical support. •Services promoting interactive and collaborative experiences to strengthen social bonds. •Enhanced engagement through follow-up services.
**Social Interaction**	•Full implementation of 4RC and SLATES functionalities. •Additional features such as like, follow, and review options. •Alternative communication services, including chat, join, call, and text messaging.
**Content**	•Display of participants’ most recent activities. •Support for multiple content formats (text, images, audio, video). •Mechanisms for participants to interact with social content (e.g., reviews, ratings). •Encouragement of user-generated content through feedback loops and engagement tools (e.g., 'like' button). •Content management mechanisms that allow users to moderate or limit discussions around specific topics.

**Table 4.** Functional social features of s-commerce. ^26^

Feature category	Functional features
Develop self-identity and sense of community	Blog pages, social login button, social user profiles, live chat tools, tag button, follow button, and discussion forums.
Promote and attract others	Like button, share button, commenting, rating tool, review tool, and recommendation tool.
Generate content	Add/update product tools, post media tools, and advertising tools.
Develop connectivity	Co-shopping and co-browsing tools.

The features highlighted in Table 3 demonstrate the multifaceted nature of s-commerce platforms. The participants and community elements emphasize the human and collaborative aspects, ensuring inclusivity, interactivity, and social bonding. Social interaction features expand these capabilities by integrating both traditional and advanced communication mechanisms. Meanwhile, the content element anchors the platform experience by promoting user-generated contributions and enabling effective content management. Collectively, these building blocks underscore the fact that s-commerce platforms extend far beyond traditional e-commerce by embedding social dynamics into every layer of platform design. This reinforces the argument that systematic RE is essential to ensure completeness, scalability, and alignment with user expectations.

ii. **Social features are based on the four S-Commerce layers**

**Figure 1.**
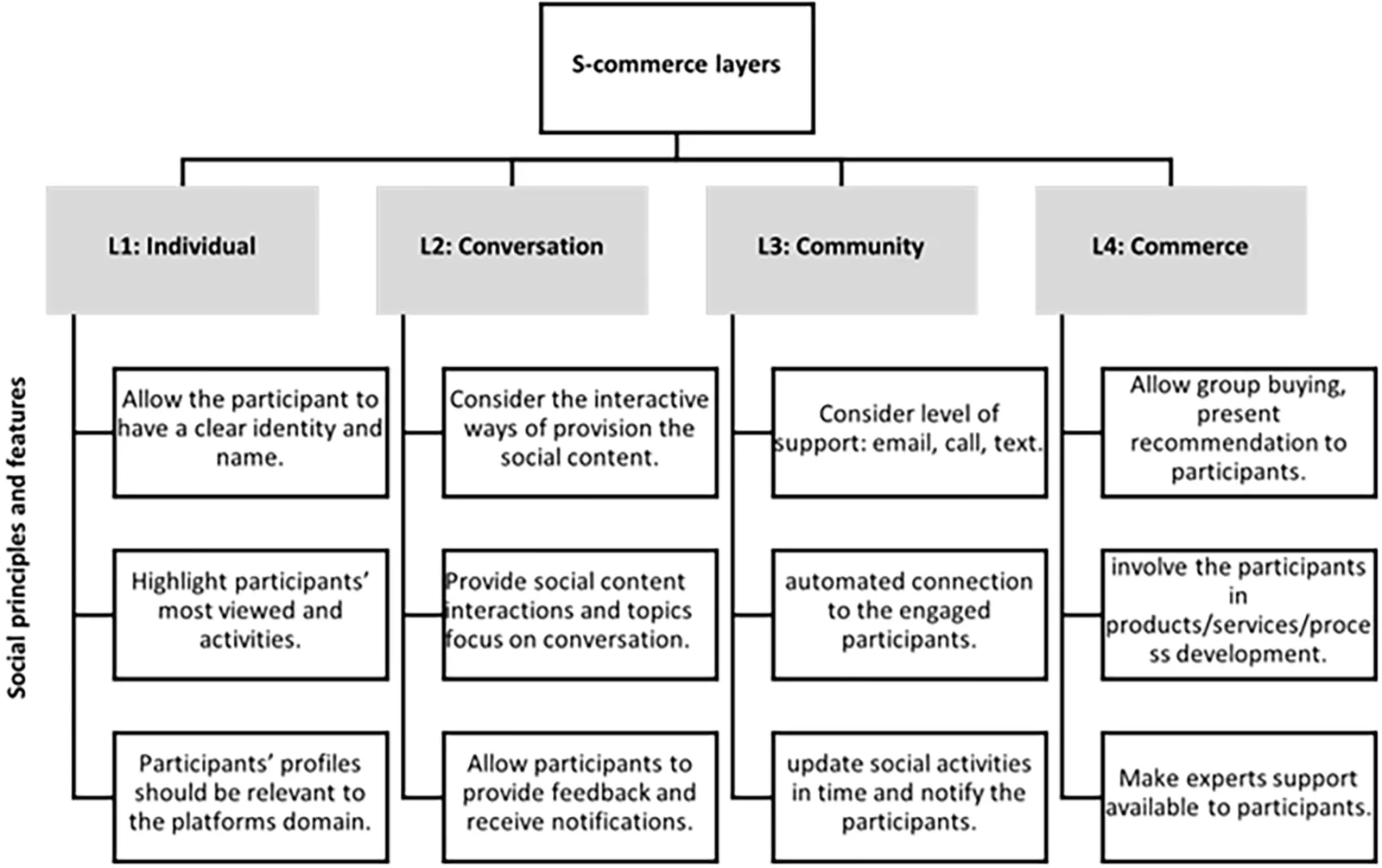
Social features Of S-Commerce layers. ^10, 27, 30^

In Refs. 10, 27, 30 the authors classified social features according to four primary s-commerce layers: individual, conversation, community, and commerce. Figure 1 presents an overview of these layers and their associated features.

• The individual layer emphasizes the importance of user identity and profile relevance, highlighting activities, ensuring clear participant identification, and enabling tailored interactions within the platform domain.
• The conversation layer focuses on interactive content provision, feedback mechanisms, notifications, and social interactions centered on dialogue.
• The community layer underscores support and collaboration, including automated participant connection, activity updates, and multiple support channels such as email, call, and text.
• The commerce layer integrates business-oriented features, such as group buying, co-designing products or services, expert support, and recommendation systems, which directly link social engagement with commercial activities.

This layered model demonstrates how social features can be systematically organized to capture the progression from individual engagement to collective community building and, ultimately, to commercial value creation.

iii. **Social features based on social design principles**

Social design principles play a pivotal role in influencing customer decision-making within s-commerce environments. Among the most widely discussed are sociability, functionality, usability, and user control, which directly impact how participants engage with platforms. Table 5 summarizes selected social features associated with these principles, as identified in prior studies. ^35, 38^ These principles collectively ensure that s-commerce platforms not only support effective transactions but also foster trust, usability, and participant empowerment.

**Table 5.** Social features based on social principles. ^35, 38^

Design principle	Social features
Sociability	Social presence through user-generated and shared content, online communities, and participant feedback mechanisms (e.g., comments and reviews).
Social Damage	Platforms should actively prevent harm to participants and communities, including inappropriate interactions, misinformation, or exploitation.
Functionality	Enabling transaction capabilities, ensuring high information quality, and guaranteeing system availability.
Usability	Delivering consistent content design, intuitive navigation, and overall ease of use.
User Control	Providing participants with robust data control mechanisms, including privacy settings and content management.

Based on the SMS and SLR, the following gaps were identified:

• Most studies emphasize design perspectives, neglecting the full RE lifecycle.
• Existing models are descriptive and omit systematic processes for elicitation, specification, validation, and management.
• The requirements analysis phase is consistently missing, undermining robust system design.
• Back-end requirements (e.g., scalability, security, integration, service quality) are largely ignored compared to front-end social features.
• Social features are implemented in fragmented and inconsistent ways across platforms.
• No comprehensive guideline or framework exists to consolidate generic requirements and tailor them into platform-specific sets using variability mechanisms.
• The transformation from e-commerce to s-commerce remains underexplored and lacks structured methodologies. ^3^

Collectively, these gaps underscore the need for a variability-driven RE framework that integrates both front-end and back-end requirements, supports systematic analysis and specification, and provides developers with actionable guidance for building adaptable, reliable, and scalable s-commerce platforms.

### 4.2 Objectives of a solution

The main objective of this study is to establish a comprehensive RE framework that supports developers throughout the RE lifecycle. Specifically, the framework is designed to:

• Generate a set of common requirements from a knowledge base of generic requirements.
• Specify requirements using natural language or UML use case diagrams.
• Negotiate, validate, and manage requirements effectively.
• Finalize platform-specific requirements by applying an instantiation mechanism.
• Enhance current development process models with a systematic RE process, addressing the well-documented problem of project failure due to insufficient RE practices.

### 4.3 The design of the framework (the Build)

We have sketched out the generic set of social requirements of the s-commerce platform and show:

• ‘What’ (The form of the framework).
• ‘Why’ (The significance of the framework).
• ‘How’ (To use the generic framework to generate or to recommend an instantiation of the requirements for a specific platform).

**First: The form of the framework**

**Figure 2.**
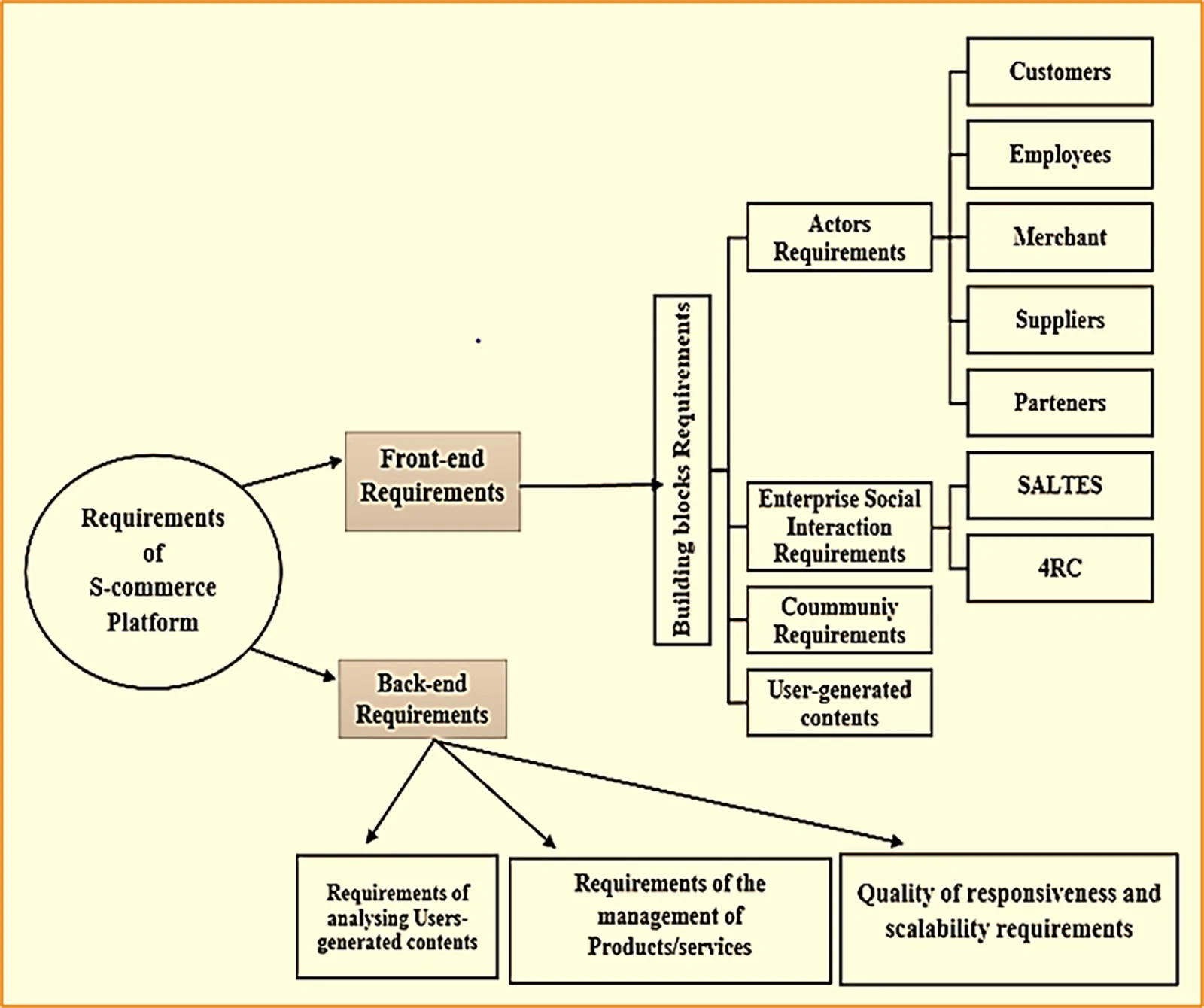
Structure of front-end and back-end requirements.

The diagram shows a hierarchical classification of s-commerce requirements into two categories: front-end requirements (participants, enterprise social interactions, community, and user-generated content with SLATES and 4RC) and back-end requirements (content analysis, product/service management, scalability, and privacy/security), highlighting the dual focus on social features and technical infrastructure. Figure 2 shows the classification of the requirements in the framework knowledge base.

***Front-end requirements***

Front-end requirements primarily concern the interactions between the platform and its users. These requirements focus on interactive social features, most notably the 4RC functionalities. The framework’s knowledge base includes 28 front-end requirements for s-commerce platforms.

Requirements are formally documented using FDL as shown in Table 6, and UML as presented in Table 7 to ensure standardization and traceability.

**Table 6.** Sample of front-end requirements using FDL in the knowledge base.

Rid	Statement	Description	Preconditions	Postconditions	Basic flow	Alternative flow	Exception flows	Social feature type
R1	As a participant, I want to be able to register on the platform using the registration form, so I can log in to the platform.	This feature enables participants to create a social profile.	The participant has the correct email address to receive the confirmation.	The participant has received confirmation from the authorized system.	1. The authorized system provides a registration form. 2. Participant should fill out the form based on the required details. 3. Participant should follow the rules of each detail. 4. The participant submits the form through the system. 5. Authorized system verifies the form and confirms it. 6. Authorized system sends the confirmation through the participant's email. 7. Participant uses the created username and the password to log in.	If the participant doesn’t receive the confirmation, he/she sends an email to the technician support team.	If the participant could not be able to submit the registration form, an error message is displayed.	Participant

**Table 7.** Sample of front-end requirements using UML in the knowledge base.

RNo.	Use case name	Use case scenario	UML Use case diagram
R1	Create a participant account	1. The authorized system provides a registration form. 2. The participant fills the form based on the required details. 3. The participant should follow the rules of each detail. 4. The participant submits the form through the system. 5. The authorized system verifies the form and confirms it. 6. The authorized system sends the confirmation through the participant's email. 7. The participant uses the created username and the password to log in.	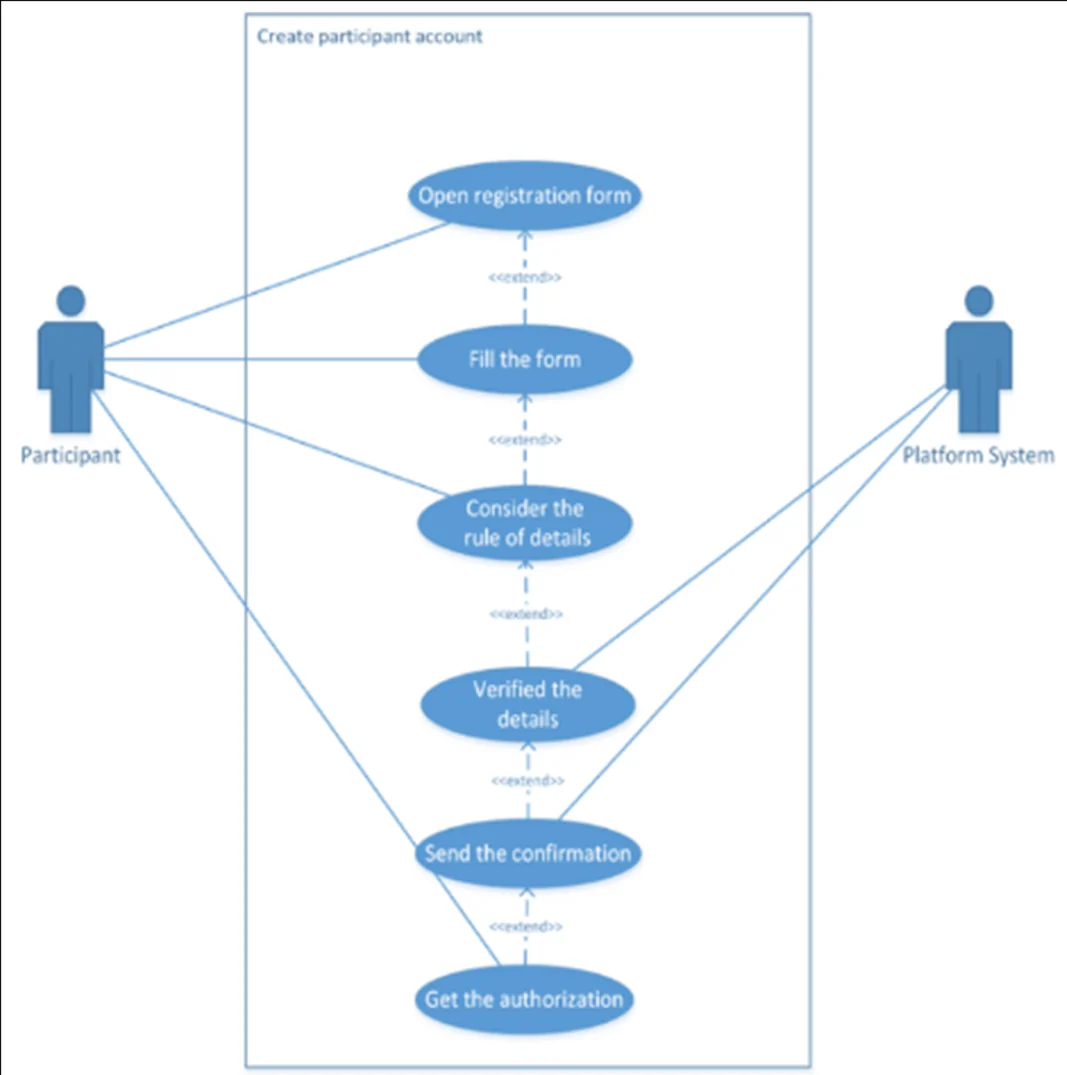

The front-end requirements are organized into four main categories: Participants, Social Features, Community, and Content. The knowledge base is dynamic and extensible, enabling developers to add new requirements while maintaining compliance with FDL and UML structures. The completed table of the front-end requirements is available at the link provided on the “Data Availability Statement”.

***Back-end requirements***

The framework’s knowledge base comprises 15 back-end requirements that ensure the platform operates smoothly, securely, and efficiently behind the scenes. These requirements involve multiple actors such as system administrators, database managers, support personnel, and analytics teams who collectively contribute to platform stability, scalability, and performance monitoring. Examples of the back-end requirements structure are presented in Table 8. The completed table of the back-end requirements is available at the link provided on the “Data Availability Statement”.

**Table 8.** Sample of back-end requirements in the knowledge base.

RNo.	Requirements statement	Explanation	Technology/Implementation
B5	The platform should be able to implement information security.	User data must be protected through encryption, secure access, and privacy controls.	Secure Sockets Layer (SSL) encryption, Hypertext Transfer Protocol Secure (HTTPS), firewalls, and access controls.
B6	The platform should support participation control.	Administrators should manage who can participate or access certain features (e.g., content moderation).	Role-Based Access Control Systems (RBAC).

**Second: The significance of the framework**

Developers of s-commerce platforms must address evolving and increasingly sophisticated requirements. To this end, the proposed framework: (i) guides developers in building platforms that align with specific requirements, social features, and customer expectations; (ii) enables a stronger focus on social features, thereby fostering customer loyalty and enhancing the enterprise’s economic performance; and (iii) outlines a comprehensive set of generic functionalities, quality attributes, and constraints that span both the front-end and back-end layers of s-commerce.

**Third: How to use the framework**

The framework is outlined as a set of generic requirements that need to be instantiated by the developer to generate the requirements of a specific s-commerce platform. This instantiation concept provides:

• A tool that helps the developer to generate a set of requirements, taking into consideration the variability of the s-commerce platform.
• A tool for recommendations that allows the developer to propose new requirements that are not included in the knowledge base (generic set).

### 4.4 The design of the framework (the Evaluate)

There are different techniques to evaluate the artifact, we have chosen the following:

A. Practical Implementation via a Web-based Platform as a prototype
B. Meta-criteria-based Evaluation
C. Expert’s evaluation (Central Information System Developers) from Sultan Qaboos University

A. **Prototype: A Practical Implementation via a Web-based Platform**

The proposed framework is structured as a generic set of requirements that developers must instantiate to generate the specific requirements of an individual s-commerce platform. This instantiation mechanism provides two key advantages:

• **Requirement generation tool**: It enables developers to derive a tailored set of requirements while accounting for the inherent variability across different s-commerce platforms.
• **Recommendation tool:** It allows developers to propose new requirements that may not yet exist in the knowledge base, thereby enriching and extending the repository of generic requirements.

**Figure 3.**
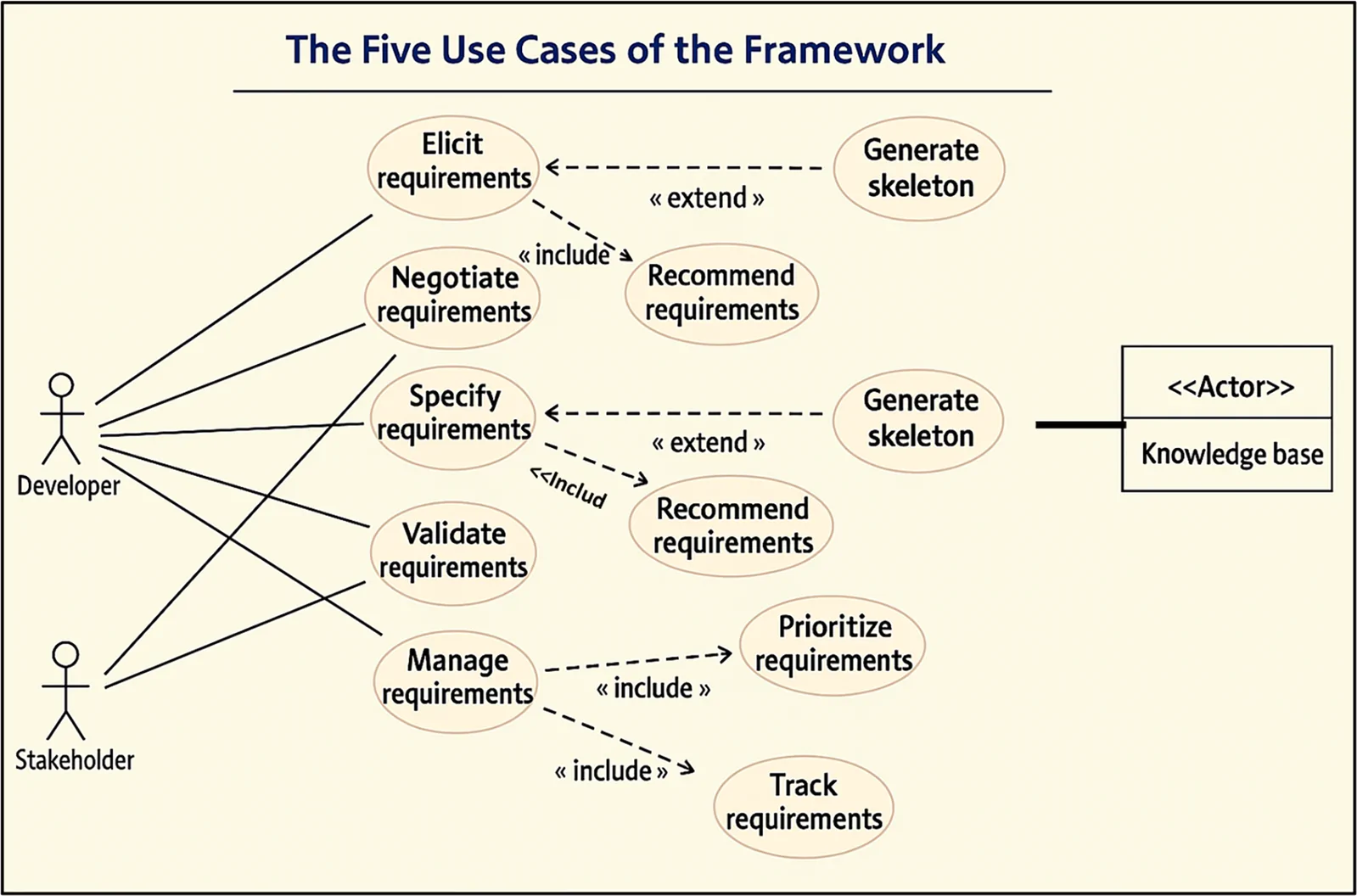
Use cases of our proposed framework.

As illustrated in Figure 3, the developer within the proposed framework can implement five core use cases: eliciting requirements, negotiating requirements, specifying requirements, validating requirements, and managing requirements. These use cases are supported by a dynamic platform underpinned by the knowledge base, which functions as a structured repository of requirements expressed in a defined elicitation and specification language. The framework defines three categories of actors:

• *Primary actor*: The developer, who actively engages with the framework to instantiate and manage requirements.
• *Secondary actor*: The platform (system), which supports the developer by hosting the knowledge base and executing instantiation processes.
• *External users:* Including enterprise staff and public users who interact with the resulting s-commerce platforms to carry out both commercial and social activities.

**Requirement generation tool and variability concept**

The variability of the s-commerce platforms, as not all the platforms are similar, (i.e., different markets have different platforms and different requirements set).

The variability mechanism within the framework is initiated through three fundamental selection dimensions: (a) requirements type selection, (b) domain type selection, and (c) social feature selection.

By incorporating structured points of variability, the framework allows developers to make deliberate choices that align with stakeholder expectations, business goals, and domain-specific constraints.

**Figure 4.**
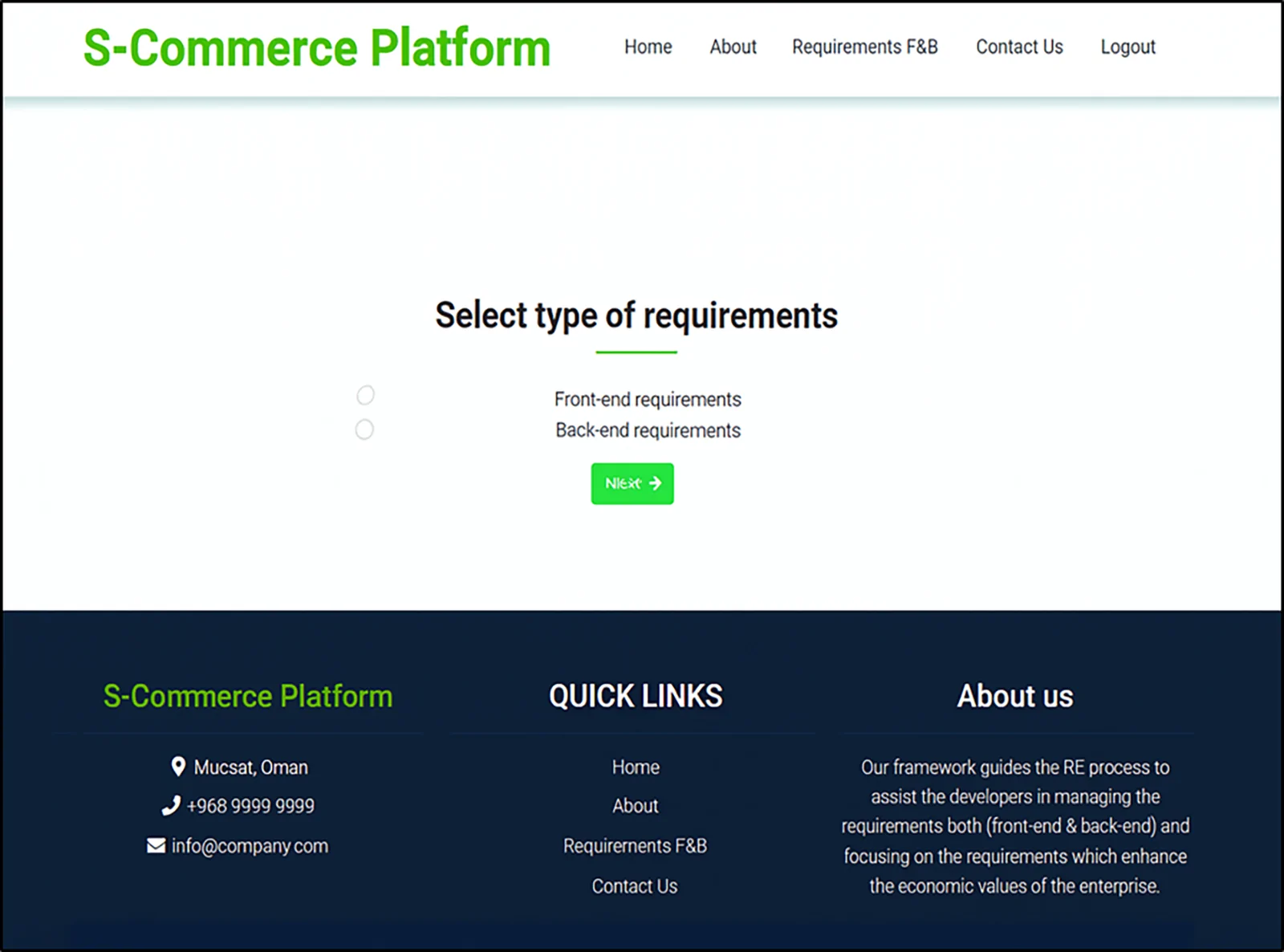
Requirement type selection.

Figure 4 illustrates the interface of the proposed s-commerce platform, where developers are prompted to specify the type of requirements they wish to manage. The interface provides two options front-end requirements and back-end requirements, selectable through radio buttons.

**Figure 5.**
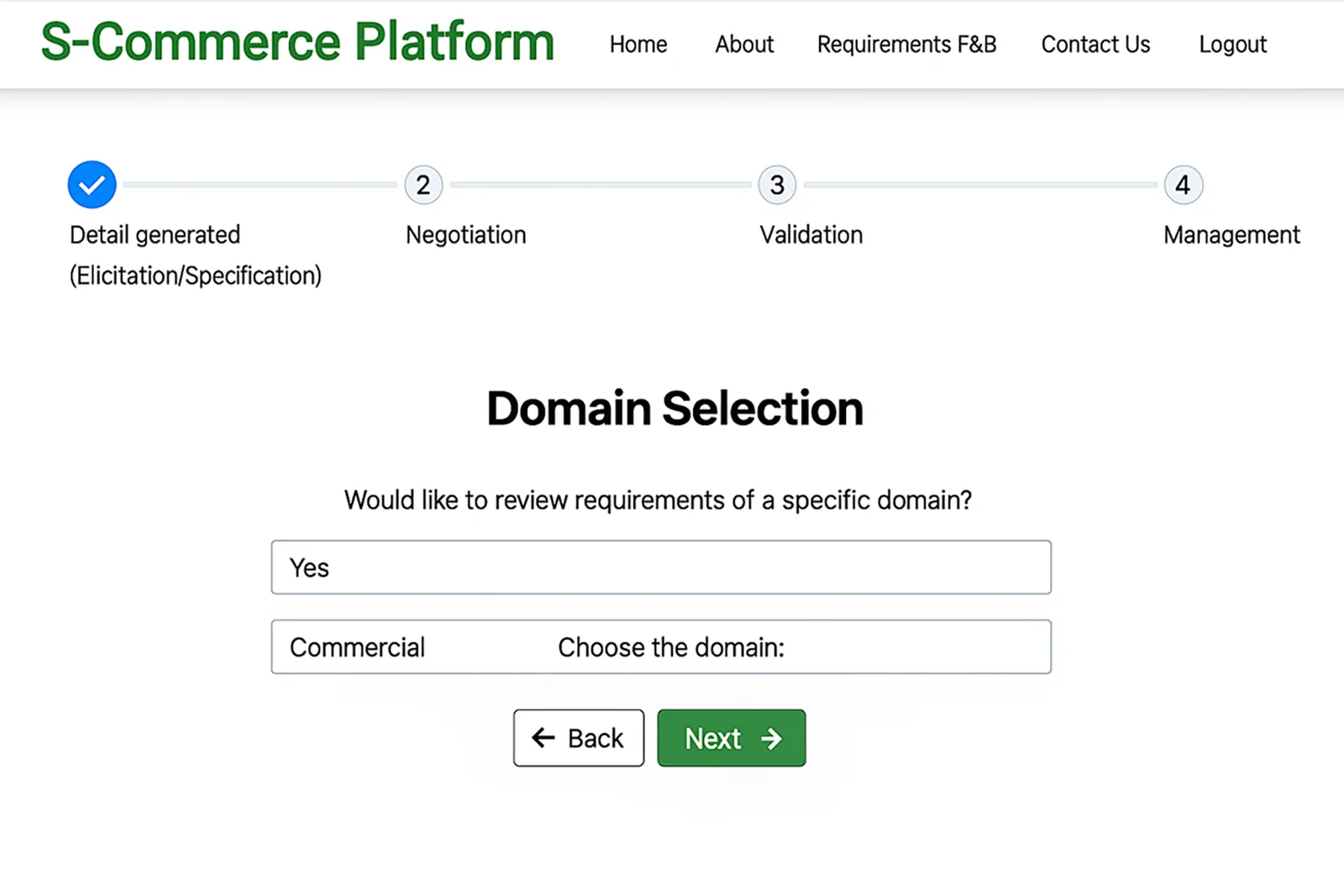
Domain type selection.

**Figure 6.**
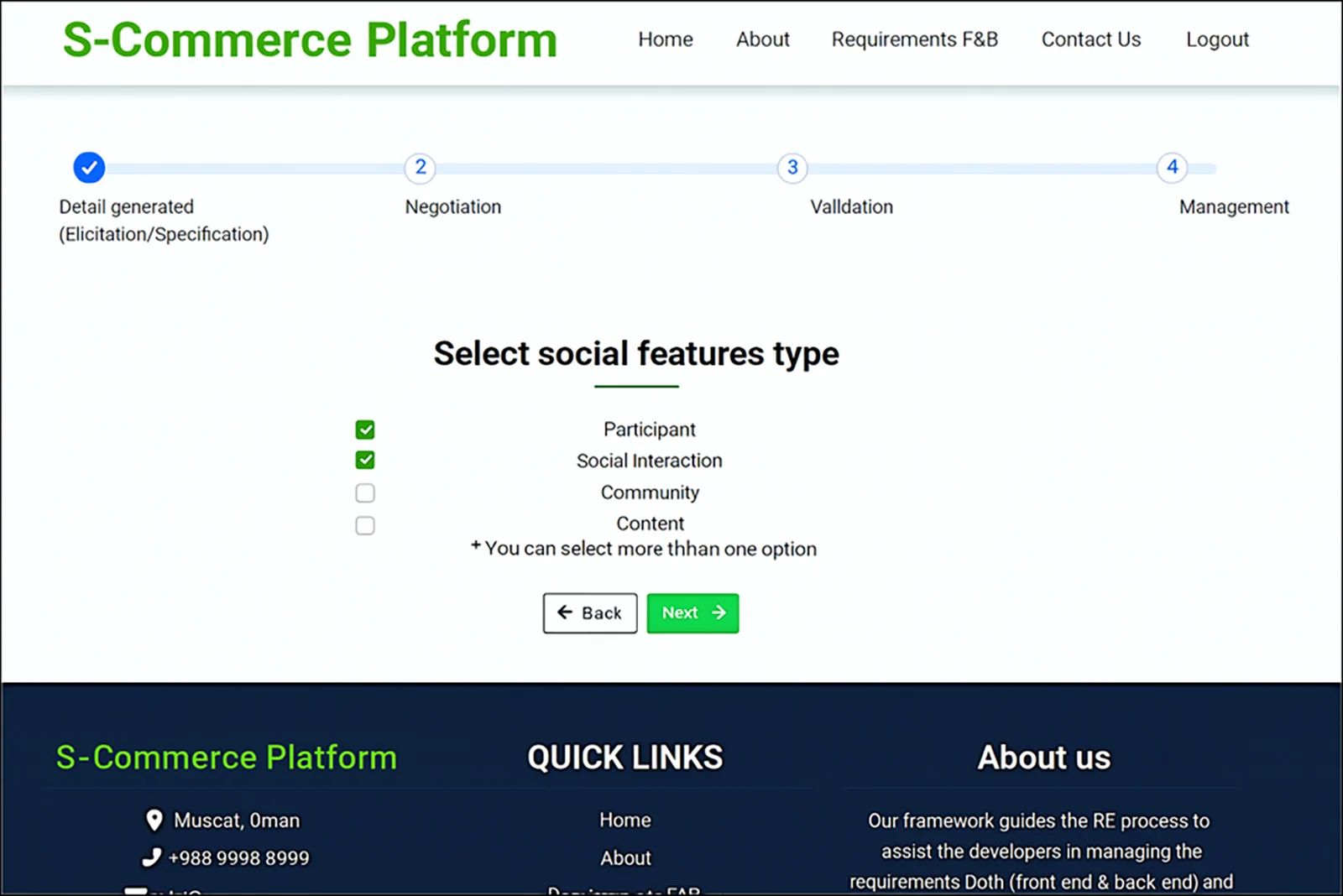
Social features type selection.

After choosing the type of requirements (front-end or back-end), the developer is prompted to select the domain type (e.g., commercial, educational, or healthcare) as Figure 5 shows, which ensures that requirements are tailored to the specific application context. In the subsequent step, the developer must also select the relevant social feature types, including participant, social interaction, community, and content. Multiple selections can be made simultaneously, enabling developers to capture the variability and richness of s-commerce platforms, as displayed in Figure 6.

This staged selection process covering requirement type, domain type, and social feature type ensures that the framework dynamically guides the developer through a structured instantiation of requirements. It thereby supports the systematic generation of context-specific requirements aligned with both platform needs and user expectations.

From the Software Product Line (SPL) perspective, the framework manages variability by distinguishing common and variable requirements across domains, promoting reuse while allowing customization. Focusing on one domain at a time improves traceability, validation, and adaptability. Variability is addressed at three levels:

1. **Requirement type selection**
  • Variation Point: Front-end vs. Back-end requirements.
  • Purpose: Guides developers to the desired requirement category.
  • SPL Role: Separates user-facing and system-side features.
2. **Domain selection**
  • Variation Point: Commercial, Educational, Enterprise, etc.
  • Purpose: Filters requirements for the chosen domain.
  • SPL Role: Combines reusable core features with domain-specific needs.
3. **Social feature selection**
  • Variation Point: Participant, Social Interaction, Community, Content.
  • Purpose: Configures social functionality per domain.
  • SPL Role: Supports variability and multi-product configuration.

After these three steps, the framework’s main requirements encompass the full range of activities within the RE process. Within this framework, the developer can access and manage all requirements, each of which is organized as follows:

1. **Consistently elicited**

**Figure 7.**
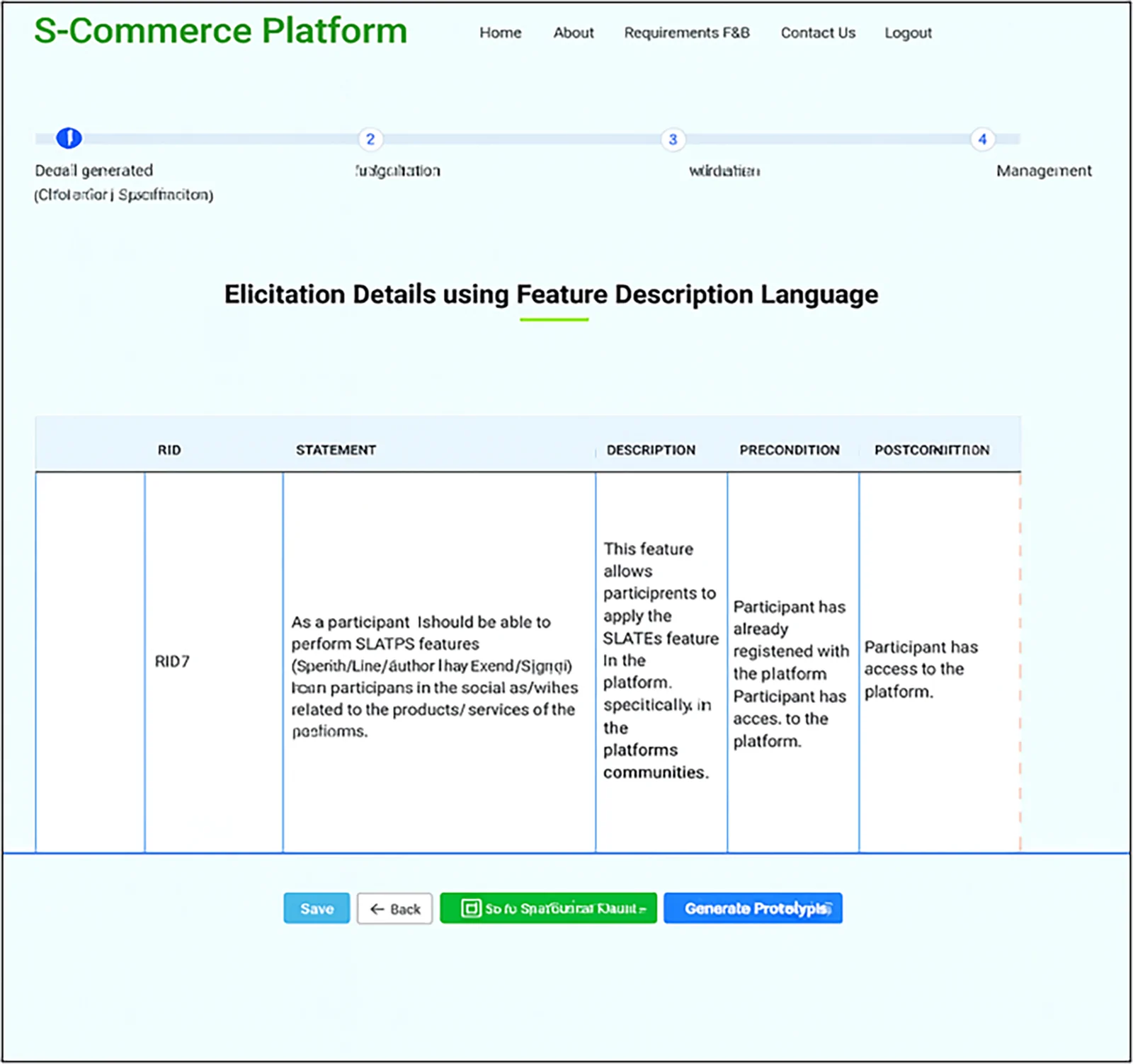
Elicitation process interface.

Requirements in the proposed framework are consistently elicited through the use of a structured platform that incorporates a predefined set of generic requirements. These requirements were formalized and recorded using the FDL, which enables clear representation of both functional and non-functional aspects. All of the 28 front-end follow the same standard of FDL. The interface of the elicitation is illustrated in Figure 7.

2. **Specified in using UML**

**Figure 8.**
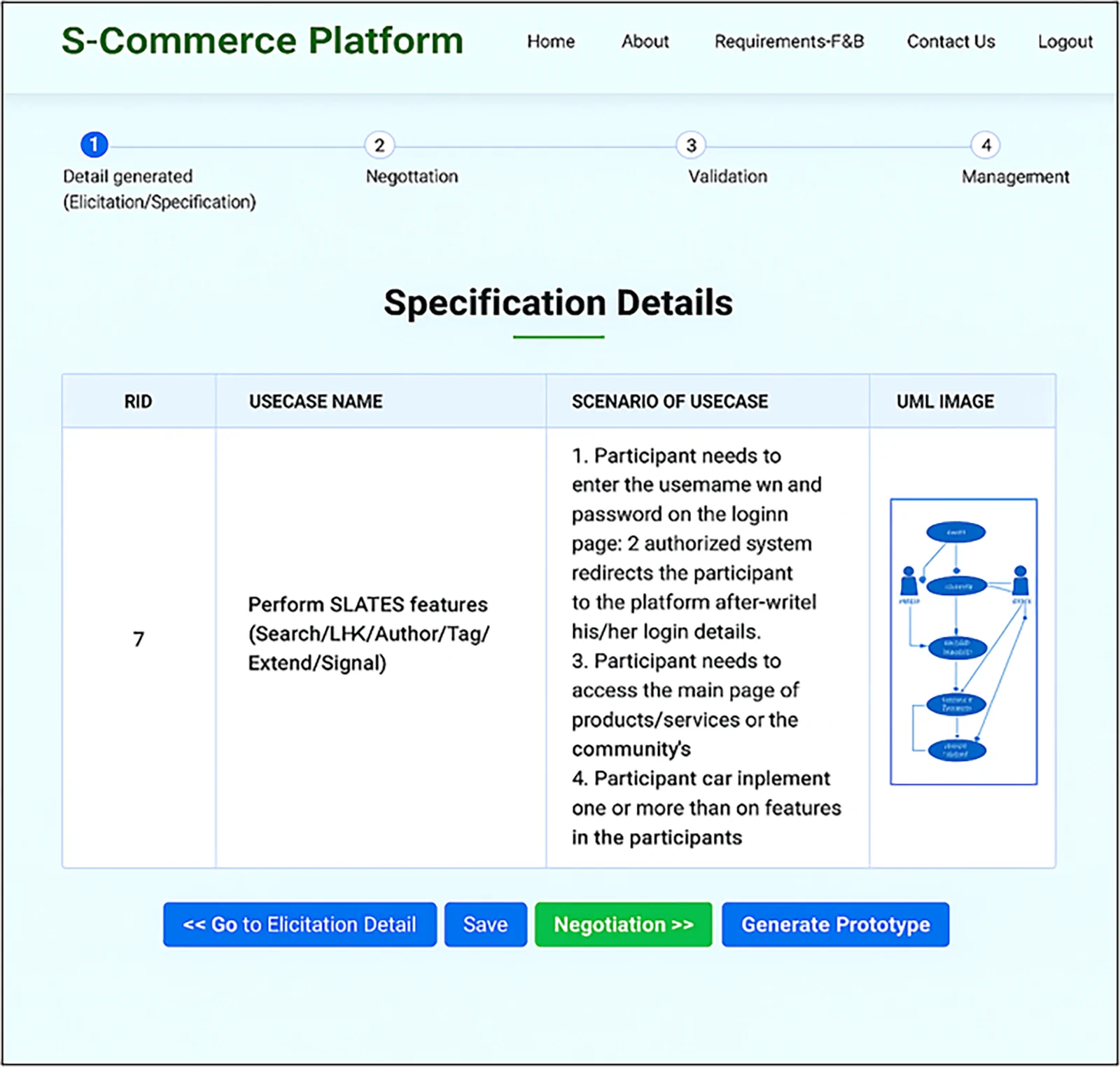
Specification process interface.

After the developer reviews the elicitation details using FDL, the system transitions to the specification stage, where requirements are formalized using UML. At this stage, the elicited requirements are transformed into structured use case representations, as illustrated in Figure 8.

3. **Generate prototype option**

Developers can proceed to the “Generate Prototype” option during either the elicitation or specification activities. This feature automatically generates a prototype website with a set of default products, while the front-end requirements included in each prototype vary according to the developer’s prior selections. This demonstrates the variability-driven approach of the framework, where prototypes are dynamically tailored to individual developer choices.

**Figure 9.**
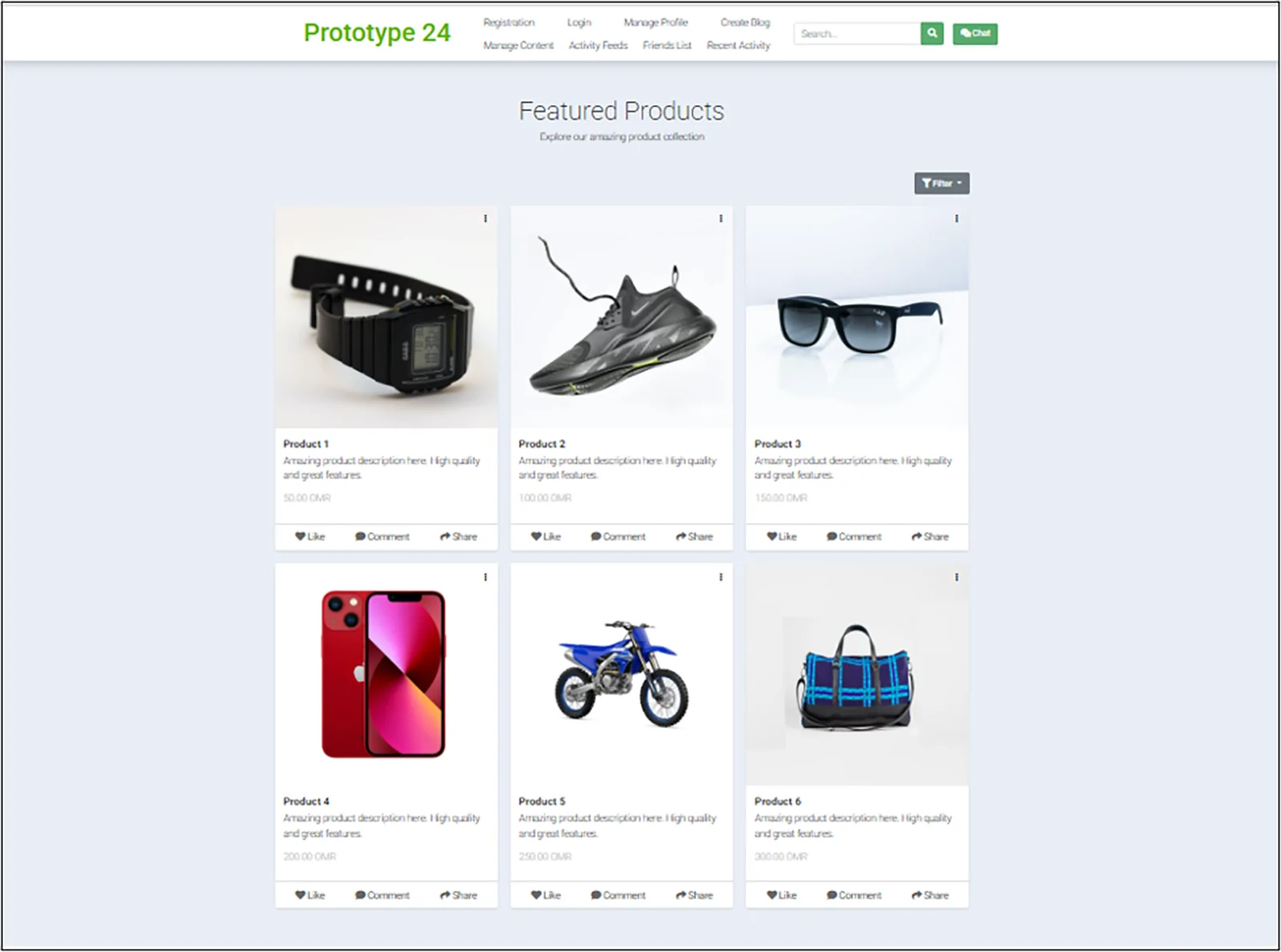
Generate prototype interface.

Figure 9 presents an example of a generated prototype, highlighting how developer inputs directly shape the platform’s structure, interaction features, and overall functionality. The ability to rapidly generate prototypes not only validates the selection of requirements but also provides immediate feedback on how social features and system elements are realized in practice.

4. **Consistently negotiate, validate and manage**

**Figure 10.**
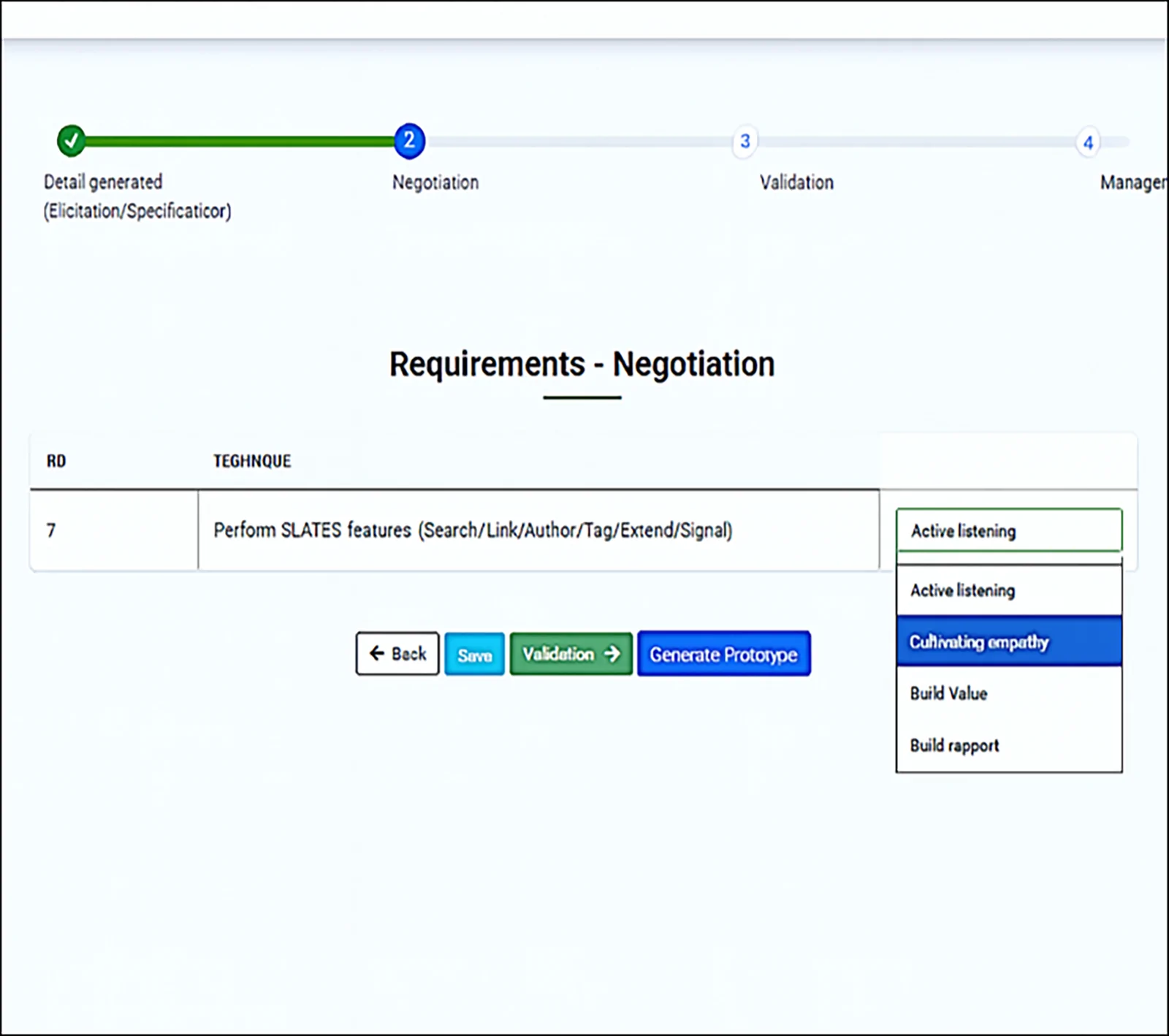
Negotiation process interface.

**Figure 11.**
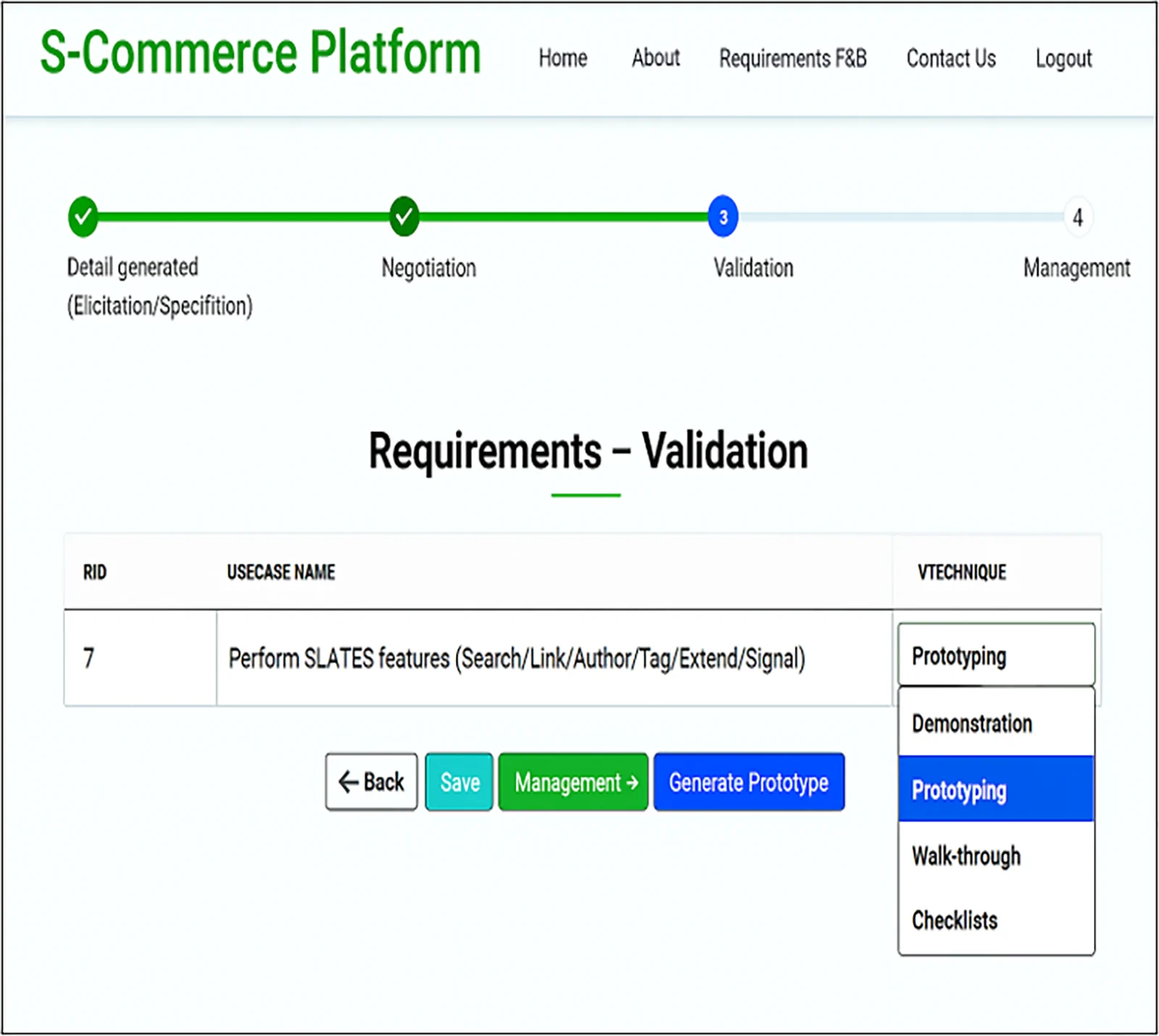
Validation process interface.

Once the specification activity is completed, developers advance to the subsequent RE activities, namely Negotiation, Validation, and Management. In the negotiation phase, developers may employ techniques such as active-listening, building value, or stakeholder discussion sessions to resolve conflicts and reach consensus on the selected requirements. During validation, approaches such as prototyping, scenario-based reviews, and checklist-based evaluations are used to confirm that the specified requirements are both feasible and aligned with stakeholder expectations, as illustrated in Figure 10 and Figure 11.

**Figure 12.**
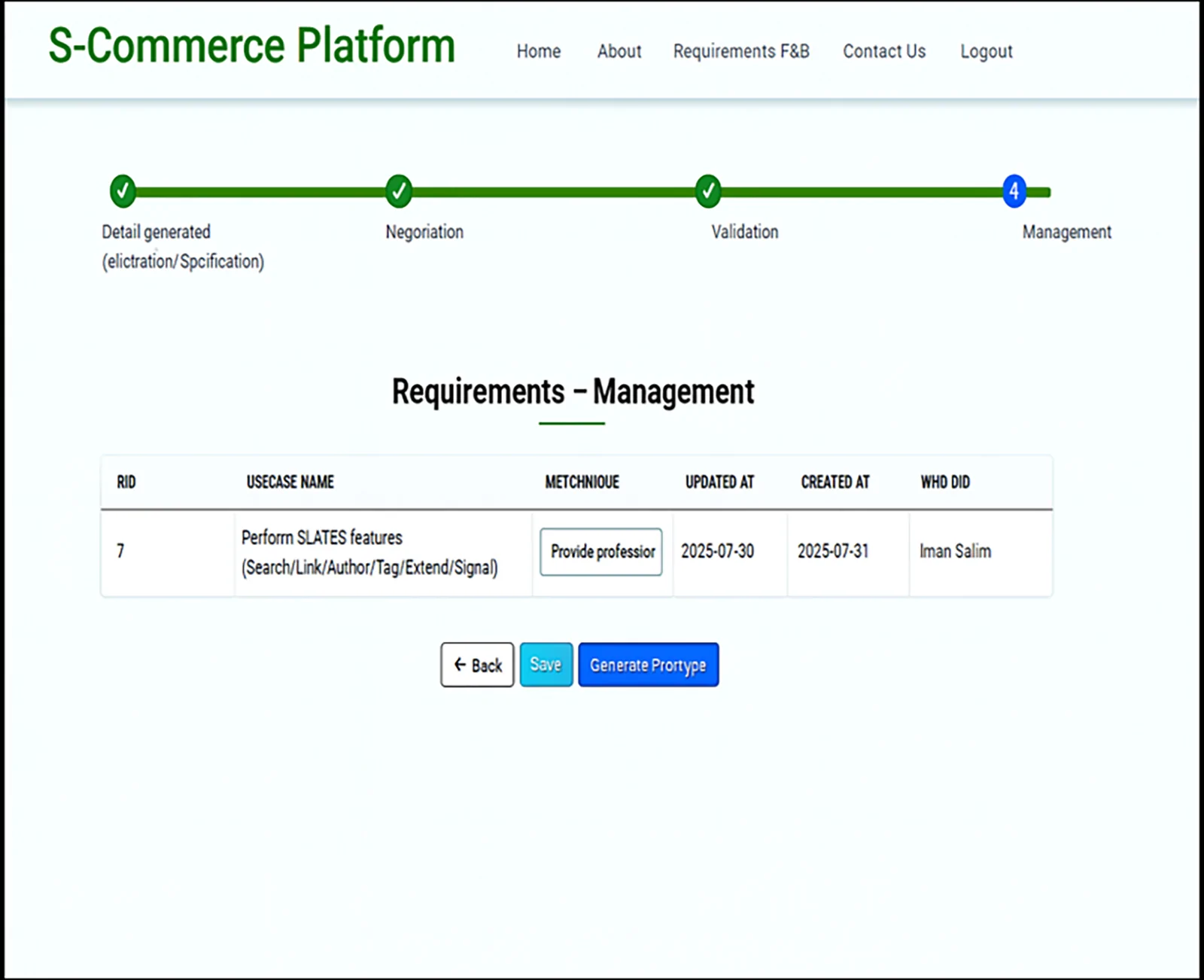
Management process interface.

The management activity then focuses on the continuous oversight of requirements throughout the project lifecycle. Developers are expected to update requirement records and apply techniques such as traceability matrices, version control, and impact analysis to ensure proper monitoring and adaptability over time. This process is essential for maintaining consistency with project goals and accommodating evolving business or user needs. Figure 12 shows the interface for the management activity, emphasizing its critical role in sustaining requirement traceability and long-term lifecycle support.

**Second: Requirement recommendation tool**

**Figure 13.**
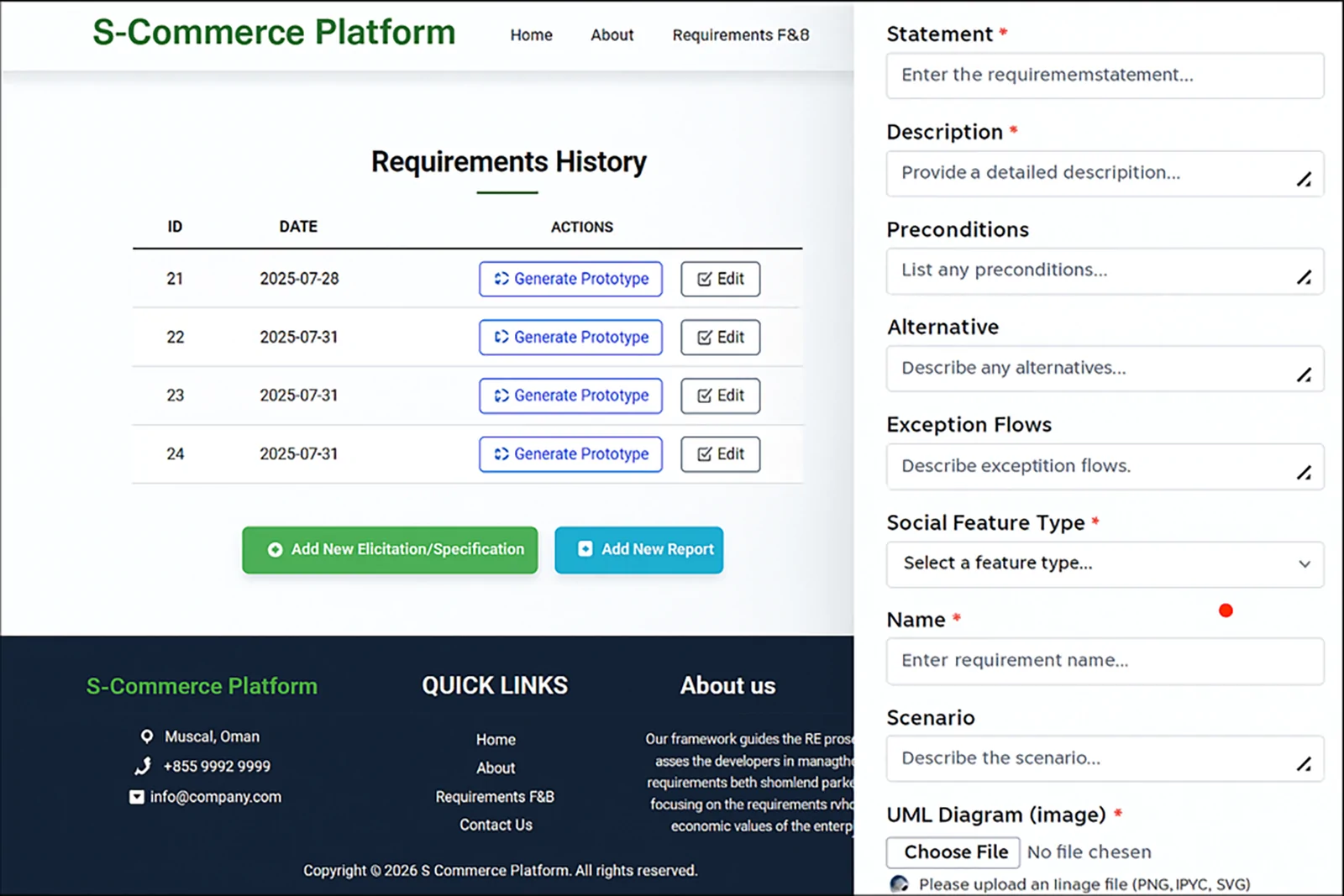
Add a new report and requirements history interface.

As illustrated in Figure 13, developers are provided with the option to add new requirements. To ensure consistency, they must specify the details of each requirement according to the criteria defined by FDL and UML, as shown in Figure 13. These details are captured simultaneously and stored in separate database tables, corresponding to their respective models.

The tool offers several key functionalities:

• Recommendation of additional requirements based on similarity analysis, usage trends, and previous configurations.
• Support for custom requirement entry, ensuring traceability to rationale and stakeholder input.
• Adaptive learning from developer choices, improving the accuracy of future suggestions.
• Annotation, tagging, and prioritization of proposed features for better organization and decision-making.

By enabling the exploration of requirements beyond the predefined catalog, the recommendation engine promotes innovation, flexibility, and customization. This is especially valuable in emerging or niche s-commerce domains, where conventional requirement sets may fail to capture unique user behaviors, regulatory considerations, or integration needs.

It is important to emphasize that any new requirements manually added by the developer are not automatically displayed as features on the prototype page. This page is strictly reserved for features derived from the predefined generic requirements list integrated into the framework. Manually added requirements are instead stored within the system for reference, documentation, or future analysis, but they do not directly populate the generated prototype interface.

Furthermore, Figure 13 shows an option of the “Requirements History” interface of the proposed s-commerce platform. This component provides developers with a transparent log of their RE activity sessions. Each record in the history corresponds to a distinct elicitation or specification instance, enabling developers to revisit prior sessions, regenerate a prototype, or update requirement selections. This mechanism strengthens traceability, accountability, and version control, ensuring that previous decisions remain accessible for review, refinement, or reuse.

Collectively, the separation between generic and manually added requirements and the integration of the requirements history interface provides a balance between stability and flexibility. While the prototype ensures consistency by relying only on the generic requirements, the history mechanism ensures adaptability by documenting new ideas, supporting iterative refinement, and maintaining full traceability throughout the RE lifecycle.

### 4.5 Back-end requirements

**Figure 14.**
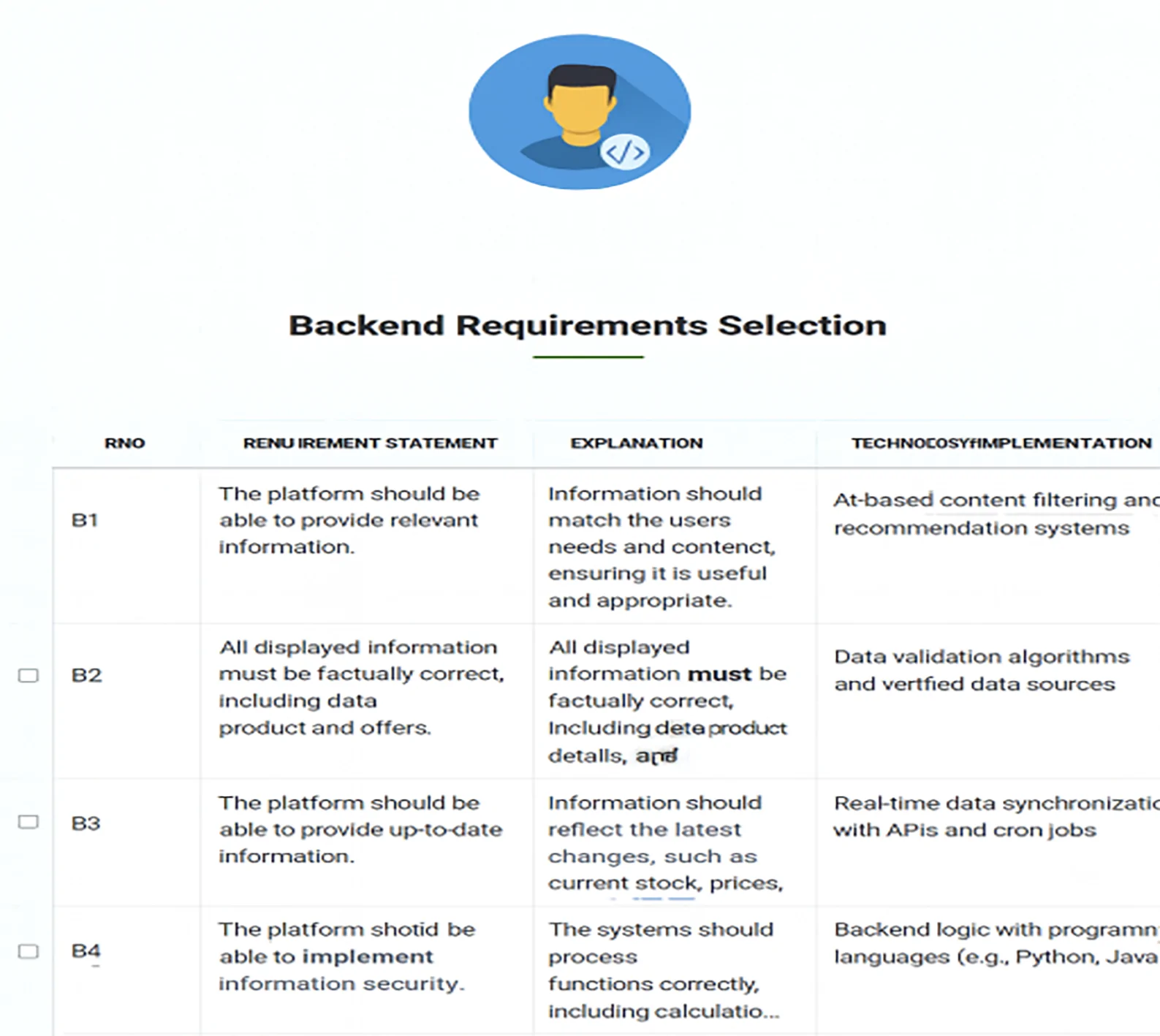
Back-end requirements interface.

When developers select the back-end option rather than the front-end, they do not navigate through the complete set of detailed RE activities. Instead, they are directed straight to the back-end requirements interface, which presents a structured list of predefined requirements, as illustrated in Figure 14. Unlike front-end requirements, which can be immediately instantiated and visualized in the prototype, back-end requirements are not directly reflected in the prototype interface.

In essence, the back-end requirements selection page enables developers to concentrate on system integrity, data governance, computational accuracy, and security assurance. While less visible than front-end features, these back-end components form the critical backbone of s-commerce platforms, ensuring that the system’s hidden architecture aligns with and supports the broader RE activities.

### 4.6 Database structure of the developed framework

**Figure 15.**
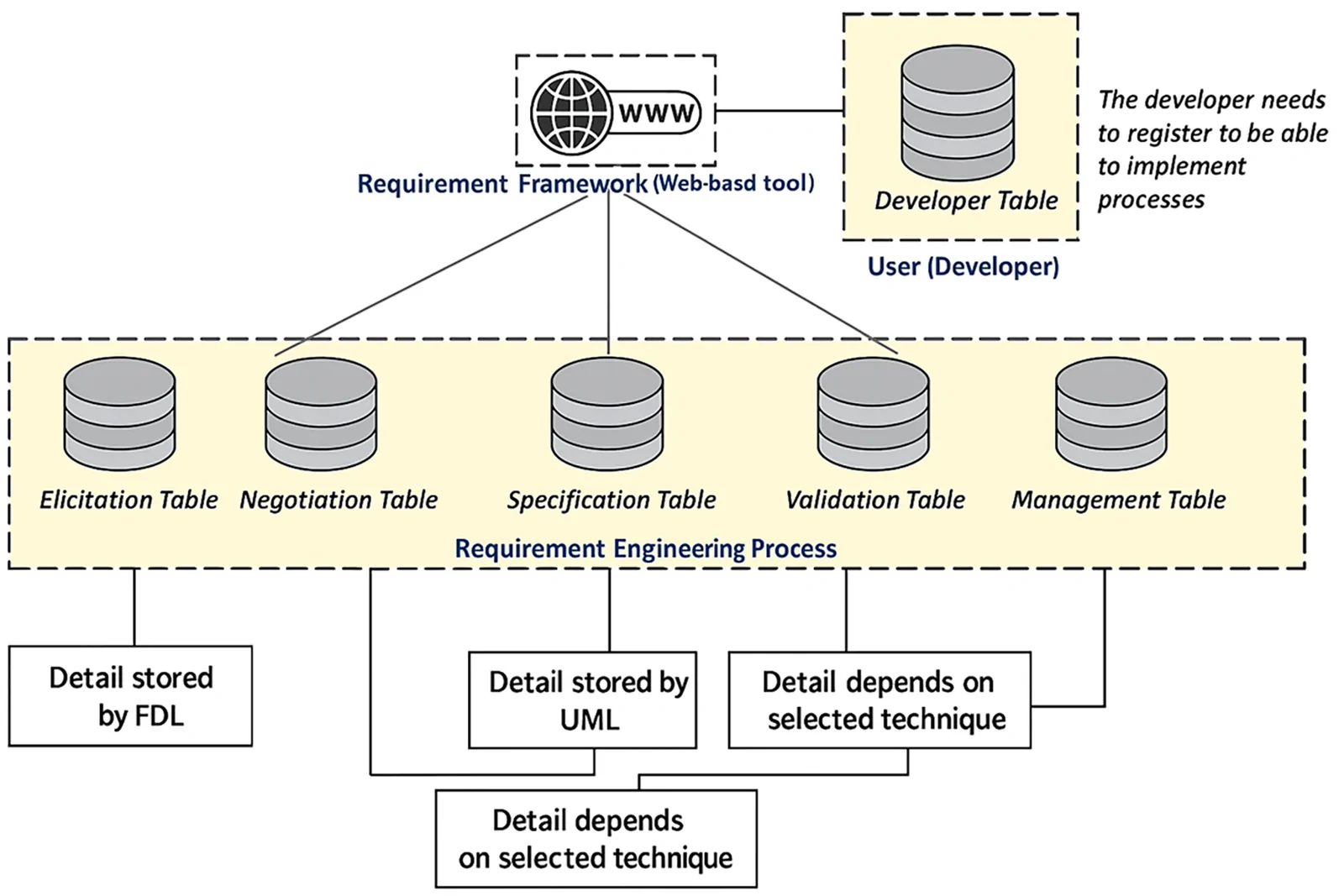
Database structure of the framework.

Figure 15 illustrates the database architecture that underpins the proposed web-based RE framework. At the core, the system is driven by a set of specialized tables, each corresponding to a distinct RE activity: elicitation, negotiation, specification, validation, and management. These tables collectively ensure that all requirement-related data is systematically captured, stored, and retrievable across the full RE lifecycle. Developers interact with the framework via the Developer Table, which maintains user registration data and links individual developers to their RE activities.

B. **Meta-Criteria-based Evaluation**

In addition to the implementation, the framework was assessed against a set of validated meta-criteria developed by. ^39^ These meta-criteria provide a structured lens for evaluating RE approaches within the context of SPLE.

The main criteria of ^39^ are:

Coverage of RE, Process Aspect, Artifact Aspect, Coverage of Variability and Commonality, Process: Domain Engineering, Process: Application Engineering, Product, Adoption Strategy and, Coverage of Tooling Support.

Table 9 shows one of the criteria and the selected framework for comparison, the full criteria table can be accessed through the link provided in the “Data Availability Statement”.

**Table 9.** Comparison between the proposed framework and other existing frameworks.

Meta-Criteria	Criteria	Our developed Framework	(J. T. Almonte et al., 2025)	(Sybella & Jönköping, 2020)	(Dąbrowski et al., 2020)	(Listyorini et al., 2020)	(Johansson, 2019)	(Ismail et al., 2017)	(Lin et al., 2017)	(Hajli et al., 2017)	(Teknologi et al., 2015)	(Wu et al., 2015)	(Huang & Benyoucef, 2015)	(Huang & Benyoucef, 2013a)	(Baghdadi, 2013)	Curty & Zhang (2013)	Huang et al. (2012)
Coverage of RE	Functional Requirements	✓	I	P	I	p	P	P	P	✗	P	P	P	✓	✓	P	P
	Non-functional Requirements	✓	✓	P	✗	✗	P	P	✗	P	✗	✗	✗	P	P	✗	✗
	Preferences	✓	✗	✗	✓	✗	P	✗	✗	I	I	✗	I	P	I	✗	✗

The comprehensive comparison, presented in the table below, synthesizes the capabilities of each framework across 28 sub-criteria. The analysis confirms that the proposed RE framework makes a timely and meaningful contribution to s-commerce platform engineering by supporting the full RE lifecycle, including elicitation, modeling, analysis, validation, verification, and management. In addition, the framework introduces advanced mechanisms for variability modeling and reuse, while providing developer-oriented tools that emphasize usability, traceability, and automation, thereby enhancing productivity and long-term maintainability.

C. **Expert’s evaluation (Central information system developers) from Sultan Qaboos University**

To validate the feasibility and applicability of the proposed framework, we engaged with developers from the Central Information System (CIS) at Sultan Qaboos University (SQU). A structured interview was conducted to gather their professional insights regarding the alignment of the framework with existing institutional systems and practices. During the session, we presented a detailed proposal along with a prototype of the alumni page on the university website ( https://www.squ.edu.om/squalumni), including instructional guidelines and interface demonstrations illustrating how the system would look and operate. The evaluation highlighted both the strengths and practical considerations of implementation, ensuring that the framework not only addresses theoretical requirements but also meets the operational needs of real-world academic environments. Furthermore, the full documentation of the interview, proposal, and the Future View Guideline is made available in the data repository, with the access link provided in the Data Availability Statement.

## 5. Discussion

### 5.1 Comparison with prior work

This study aimed to address the absence of a systematic, requirements-driven framework for developing of s-commerce platforms. While previous research has proposed conceptual models, layered architectures, and feature sets, ^10, 30– 32^ these approaches have mainly remained descriptive. They identified what social features exist but offered little prescriptive guidance for developers on how to manage requirements across the full RE lifecycle. By contrast, the proposed framework integrates variability management, front-end and back-end requirements, and tool-supported instantiation mechanisms, thus advancing both the research discourse and the practice of s-commerce engineering.

### 5.2 Strengths of the framework

The results of the SLR revealed that prior models, such as the four-layer architectures (individual, conversation, community, commerce), ^10, 30, 31^ provide valuable conceptual clarity, but do not embed mechanisms for requirements elicitation, negotiation, or validation. Similarly, building block–based studies ^3, 22, 33^ emphasize participants and interactions yet fail to connect these elements to structured RE processes. The framework developed in this study extends these contributions by embedding such conceptual insights into a tool-supported process that guides developers through elicitation, specification, negotiation, validation, and management.

A distinctive strength of the framework lies in its dual classification of requirements. Front-end requirements capturing SLATES and 4RC features support user engagement and social interaction, while back-end requirements ensure scalability, security, and commercial process reliability. By explicitly combining visible (front-end) requirements formatted using FDL and UML and invisible (back-end) requirements, the framework ensures both a rich user experience and a trustworthy system-level infrastructure.

From SPL perspective, the framework operationalizes variability management at three levels: requirement type selection, domain selection, and social feature selection. This structured variability supports reuse across domains while allowing customization for context-specific needs, enhancing adaptability, reducing redundancy, and improving maintainability.

The integration of tool support provides further novelty. The recommendation engine extends beyond static requirement catalogs by suggesting additional requirements based on similarity analysis, prior usage, and stakeholder input. Likewise, the prototype generator offers immediate visualization of selected requirements, enabling iterative validation of design choices. Such automated support is rare in existing approaches and represents an important contribution for both novice and expert developers.

### 5.3 Implications

The findings of this study carry several implications. For scholars, the framework demonstrates how descriptive models can be advanced into prescriptive, variability-driven, and tool-supported processes, thereby enriching the theoretical landscape of RE in s-commerce. For practitioners, it provides actionable guidance for systematically identifying, prioritizing, and implementing social features while maintaining system-level reliability. By embedding traceability, accountability, and continuous improvement, the framework enables organizations to adapt with the rapid evolution of s-commerce ecosystems.

## 6. Conclusion

The purpose of this study was to address the absence of a systematic, requirements-driven framework for developing s-commerce platforms. Existing models and feature sets have largely remained descriptive, offering conceptual classifications without providing prescriptive guidance for developers across the full RE lifecycle. To overcome this gap, the study proposed and evaluated a comprehensive framework that integrates front-end and back-end requirements, structured variability management, and tool-supported instantiation mechanisms.

The results demonstrate that the proposed framework supports the complete RE lifecycle including elicitation, specification, negotiation, validation, and management while also embedding automation through a recommendation engine and prototype generator. Compared to existing models, the framework ensures greater traceability, adaptability, and scalability, providing developers with systematic guidance that was previously lacking in earlier approaches. The evaluation further highlighted that while front-end requirements can be effectively visualized through prototyping, back-end requirements remain more dependent on technical integrations, underscoring the need for further refinement.

### 6.1 Theoretical contributions

• Dichotomized taxonomy distinguishing front-end from back-end requirements.
• Introduction of structured variability modeling for instantiation, customization, and reuse.
• Validation of framework adaptability through structured evaluation.
• Bridging RE theory with social computing practices.
• Methodological rigor via the DSRM and meta-criteria assessment.
• Academic advancement in RE for s-commerce systems.

### 6.2 Practical contributions

• Actionable framework spanning the entire RE lifecycle.
• Tooling support for instantiation using SPL, variability modeling, and recommendation techniques.
• Governance and traceability mechanisms for accountability and change management.
• Organizational resources such as templates, checklists, and training materials.
• Strategic value by enabling economic assessment and informed decision-making.

### 6.3 Future work

Despite these contributions, the framework faces limitations, particularly in visualizing of back-end requirements and relying on conceptual evaluation. Future research should therefore focus on large-scale empirical validation through case studies, experiments, and industry collaborations, as well as technical enhancements such as adaptive machine learning–based recommendation mechanisms and improved visualization of back-end requirements.

### Ethics and consent

Ethics and consent were not required.

## Data Availability

The data underlying this study, including the comprehensive table of results that integrates all front-end and back-end requirements together with the full table of the evaluation, will be made available at the DOI: https://doi.org/10.6084/m9.figshare.30121900.v1. ^40^ Access will ensure transparency, reproducibility, and further scholarly engagement with the study’s outcomes. Data are available under the terms of the Creative Commons Attribution 4.0 International license (CC-BY 4.0).
