# Supplementary material for: A Generic Requirement Engineering Framework for Social Commerce Platforms: Front-end and Back-end Features
Source: F1000Res. 2025 Oct 9;14:1059. [Version 1] doi: 10.12688/f1000research.170127.1 (PMC13560085; doi:10.12688/f1000research.170127.1)
Supplement: Peer review history

# Reviewer report: Reviewer response for version 1

**DOI:** 10.5256/f1000research.187548.r510807

**Published:** 2026-08-22

**Authors:** Camille Salinesi

**Affiliations:** 1 Paris 1 Panthéon–Sorbonne University, Paris, France

The paper « *A Generic Requirement Engineering Framework for Social Commerce Platforms: Front-end and Back-end Features »*, by Al Jassasi, Baghdadi and Al Hamdani [5], addresses an increasingly relevant problem at the intersection of requirements engineering (RE), and software product line engineering, with a very interresting application to social computing, e-commerce. The authors propose that rather than studying social commerce from the usual perspectives of consumer behaviour, technology adoption or business models, one should consider a more engineering-oriented question: *how can the knowledge accumulated about social-commerce systems be transformed into reusable requirements assets that can support the engineering of new platforms?*

 The starting point is the observation that social-commerce research has identified numerous social mechanisms, design principles and platform characteristics. However, these results remain dispersed across studies and are not necessarily expressed in a form directly usable by software designers. Starting from there, the authors show several challenges : defining a sufficiently generic body of requirements, handling commonality and variability across platforms, enabling requirements reuse; and putting all together all these elements in an engineering framework encompassing requirements elicitation, specification, negotiation, validation and management.

 Beyond the social commerce domain, the result is a generic Requirements Engineering (RE) framework developed following a design science research methodology. The framework uses literature studies to explore the domain at hand, identify a collection of features and requirements and then formalize them into a reusable repository structured with a software product line specification model. The first littérature stuy provides the core part of the knowledge from which the framework is constructed. These sources are then complemented by the extraction of features from existing platforms and by knowledge drawn from established RE approaches. The paper shows that applied to the social commerce domain, this method produces a knowledge base contains 43 well formalized reusable requirements connected to a feature model. The framework also relies on the use of variability mechanisms to transform this generic body of knowledge into requirements for a particular social-commerce system. Three principal dimensions are considered: the type of requirement, the application domain and the type of social feature..

 This conceptual framework is implemented with a web application that supports requirements elicitation, specification, negotiation, validation and management, incorporates a mechanism for proposing additional requirements, and is able to generate front-end prototypes corresponding to the selected requirements.

 The framework is evaluated through its implementation, a comparison with other frameworks using SPL-oriented meta-criteria, and feedback collected from developers at Sultan Qaboos University.

 Al Jassasi’s et al paper [5] is a stage in a research programme developed by the same authors over several years. In 2023, Al Jassasi’s and Baghdadi’s earlier work [3] explicitly restricted itself to the design of a front-end requirements framework. The central proposition was then already to establish a generic set of social-commerce requirements and to use this generic representation to generate or recommend an instantiation appropriate to a particular platform. At that time, the focus was therefore already on building reusable requirements assets and artifact-oriented RE, but the scope remained mainly front-end and design-oriented.

 Their 2025 paper [4] broadened this perspective. It explicitly described the intended framework as comprehensive and instantiable and extended the domain model to both front-end and back-end elements. The emphasis therefore moved from a front-end requirements catalogue towards a more complete representation of the technological and social dimensions of a social-commerce platform.

 The 3rd paper [5] of this line of work can be interpreted as the consolidation and operationalization of this trajectory. The reusable knowledge base is now described more concretely as a set of 43 requirements; variability is organized around explicit selection dimensions; a web-based artifact implements the proposed process; interfaces are provided for several RE activities; front-end selections can generate prototypes; and the framework is subjected to prototype-based, meta-criteria-based and practitioner-oriented evaluation.

 Consistently with the iterative nature of the design science methodology, the novelty of third paper within this sequence is therefore not a radical replacement of their preceding work. The noverlty is more incremental in that it lies in the integration of the earlier notion of a generic and instantiable catalogue is developed into a reusable requirements repository, linked to variability decisions and embedded within an executable RE environment.

 The key idea is that published knowledge about a family of systems becomes raw material for domain engineering. Recurrent concepts, features and requirements are identified across the scientific literature, consolidated into reusable requirements assets and subsequently instantiated for particular systems.

 This perspective is particularly attractive because literature reviews cease to be solely descriptive research instruments. Here, SLR or SMS are part of the process through which a domain knowledge base is engineered.

 The paper shows how these requirements can be asociated with structured descriptions such as use cases, implementation-related information. For instance, the FDL representation used for front-end requirements records a statement, description, preconditions, postconditions, basic flow, alternatives, exceptions and social-feature category. The repository is therefore intended to contain more semantic information than a simple feature checklist. In the proposed method, all these elements are drawn from litterature reviews.

 This approach opens an interesting connection between requirements engineering, domain engineering, knowledge management and software product lines. Once established, the repository can potentially serve several projects rather than a single project, and newly acquired knowledge can enrich the reusable domain assets over time.

 This idea is not entirely novel : it actually belongs to a well-established family of research questions in SPL engineering. Rather than building a product line entirely *proactively*, domain, engineers can start from existing products, specifications, source-code repositories, product descriptions or other bodies of knowledge and reverse engineer the commonalities, variations and dependencies of a family of systems.

 Five contributions are particularly interesting in that regard: Niu and Easterbrook's method to extract SPL requirements from natural-language requirements [6]; She et al.'s approach to reverse engineer feature models from existing configurable systems [7]; Davril et al.'s proposal for the construction of feature models from large collections of informal product descriptions [8]; Mefteh et al.'s technique for mining feature models from functional requirements of product variants [9]; and Dalibor et al.'s derivation of reusable variability knowledge from a systematic mapping study on Digital Twins [10].

 Together, these works illustrate substantially different answers to the same broad question: how can an implicit product family be reconstructed from evidence that already exists?

 Niu and Easterbrook [6] are particularly close to the RE dimension of the present study. Their objective is explicitly to construct product-line requirements assets by mining existing natural-language requirements specifications. They introduce *Functional Requirements Profiles* (FRPs), extracted using linguistic regularities such as verb–direct-object relationships. Semantic analysis is then used to uncover variation structures, while the merging of profiles helps identify dependencies between requirements.

 Crucially, the resulting variability is represented through an Orthogonal Variability Model (OVM) containing variation points, variants, dependencies and constraints. Thus, the contribution does not stop at identifying recurrent requirements: it explains how textual evidence is transformed into reusable assets and subsequently into a structured variability model.

 The similarity with Al Jassasi et al. lies in the intention to transform existing knowledge into reusable requirements assets. The principal difference concerns the source material and the scope of the artifact. Niu and Easterbrook mine existing requirements specifications for a family of related systems; Al Jassasi et al. primarily consolidate domain knowledge obtained through literature studies and platform analysis, and then embed it in a broader RE support environment.

 She, Lotufo, Berger, Wąsowski and Czarnecki [7] approach the problem from the feature-model side. Their work asks how a feature model can be reconstructed from information already present in configurable software systems. A central difficulty is not simply identifying features, but recovering their organization and semantics, particularly parent-child relations and other dependencies. The method was explored on substantial configurable systems including Linux, eCos and FreeBSD.

 In this work, a collection of features becomes a feature model only when the relationships among those features are made explicit. Hierarchy, commonality, optionality and configuration constraints transform a catalogue into a model on which reasoning can be performed.

 Al Jassasi et al. follow a different route, as they begin from domain requirements rather than implementation variability and are intended into upstream engineering rather than software reverse engineering. Nevertheless, the underlying problem is analogous: recovering an explicit representation of a family from evidence in which the family structure was previously implicit.

 Davril, Delfosse, Hariri, Acher, Cleland-Huang and Heymans [8] extend the reconstruction problem beyond software repositories. Their approach automatically constructs feature models from large collections of informal descriptions of products available in online repositories. Instead of requiring an existing SPL, the method exploits textual descriptions of many individual products to infer a model of the product family.

 This contribution is especially relevant because it demonstrates that product line knowledge can be reconstructed from heterogeneous and relatively informal external evidence. The conceptual pattern is close to that of the social-commerce article: a large body of descriptions contains distributed knowledge about a domain; systematic processing of this body can reveal recurrent features and variability; this information can then be consolidated into an explicit reusable model.

 The difference is again significant : while Davril et al. focus on automated feature-model synthesis from product descriptions (and thus more concerned by the automation of the extraction process), Al Jassasi et al. are more concerned with producing requirements-level domain assets that can subsequently support RE activities.

 Mefteh, Bouassida and Ben Abdallah [9] provide an even more direct bridge between requirements and product line modelling. Their method automatically extracts a feature model from the functional requirements of several product variants. Natural-language processing identifies potential features; classification techniques harmonize different names for similar features; Formal Concept Analysis distinguishes mandatory and optional features; and semantic criteria contribute to the identification of constraints and to the structure of the resulting feature model. The generated models are then assessed quantitatively, comparatively and through expert feedback.

 This comparison is instructive because it makes explicit several transformations that remain implicit in many domain-engineering approaches: requirements => candidate features => feature equivalence => commonality/optionality => structural relationships => constraints => feature model.

 Al Jassasi et al. construct a reusable domain repository before individual variants necessarily exist. Their ambition is consequently closer to knowledge-based domain engineering. Nevertheless, Mefteh et al. illustrate why the transformation rules between requirements evidence and variability models deserve to be treated as a research contribution in their own right.

 Last, Dalibor, Jansen, Rumpe, Schmalzing, Wachtmeister, Wimmer and Wortmann [10] constitute perhaps the closest methodological precedent to the distinctive idea developed in the present paper. Their cross-domain study of Digital Twins uses a Systematic Mapping Study as a domain-engineering instrument. The literature is systematically collected and classified, engineering concerns are extracted and synthesized, and the resulting common and variable characteristics are organized into feature models covering different dimensions of Digital Twin engineering.

 The resemblance is noteworthy: in both cases, scientific publications contain distributed knowledge about a category of systems, and systematic review techniques are used to transform this knowledge into a reusable representation of the domain. The goals are nevertheless different. Dalibor et al. primarily seek to characterize the Digital Twin domain and its variability, while Al Jassasi et al. seek to go one step further by turning the resulting knowledge into an operational requirements repository that developers can instantiate and manipulate through an RE tool.

 Seen against this literature, the originality of the approach does not need to be sought in the general idea of reverse engineering commonality and variability. That problem has long been part of SPL engineering. Nor does it rest solely on extracting features from textual material or on using a systematic literature review to characterize a family of systems.

 Precisely because this is the most interesting contribution, the article would benefit from considerably greater methodological detail about how this repository was actually constructed.

 The SMS and SLR are currently described mainly through their research questions and their resulting findings. The paper does not provide enough information to reproduce the reviews independently: complete search strings, queried databases, dates of searches, deduplication procedures, inclusion and exclusion criteria, screening decisions, quality assessment, coding procedures and the complete list of selected studies are not sufficiently documented. Yet these reviews are part of the evidential foundation for the 43 generic requirements.

 An even more important issue concerns the transformation from publications and observed platforms to requirements. The article states that requirements were collected through an SLR, extraction from real-world platforms and integration of existing RE knowledge, but the derivation procedure itself remains insufficiently explicit. Several methodological questions therefore remain open. What constitutes one candidate requirement? How are two differently worded requirements recognized as equivalent? How are conflicting formulations reconciled? What evidence is necessary before a requirement becomes “generic”? How are requirements traced back to the publications or systems from which they originate? How are common requirements distinguished from variable ones? How are dependencies discovered? How is completeness assessed?

 These questions are not secondary implementation details. They concern the method through which the paper's principal research artifact is produced.

 One valuable (and small) enhancement would be to enrich the associated online repository. The article already provides a Figshare dataset containing requirements and evaluation material. The repository could additionally contain the complete SMS and SLR datasets: bibliographic search results, list of screened papers, included and excluded studies, exclusion reasons, extraction forms, coding tables, etc.

 Such material would substantially strengthen both reproducibility and the scientific status of the resulting requirements repository.

 In conclusion, the contribution by Al Jassasi, Baghdadi and Al Hamdani must be understood as part of a broader movement from single-project requirements engineering towards reusable requirements knowledge. Social commerce provides the application domain, but the research idea extends beyond it.

 The  key insight of the paper is that the scientific literature and observations of existing systems can be mined to establish a reusable body of requirements and variability knowledge, which can subsequently be exploited to guide the engineering of new systems. This idea connects naturally with a substantial body of the SPL litterature on extracting requirements assets, reverse engineering feature models and reconstructing commonality and variability from existing evidence.

 This comparison does not diminish the originality of the pape ; I rather find that it clarifies it. Whereas previous approaches have reconstructed product-line assets from requirements documents, configurable systems, product descriptions, product variants or systematic mappings, this work seeks to integrate a domain-specific requirements repository, variability management and an operational RE process.

 The next methodological step is therefore clear. The transformation from literature and observed platforms to reusable requirements must itself become explicit, reproducible and traceable. The SMS and SLR protocols and datasets should be fully available; the derivation of each requirement should be documented; and the variability model should progress from a set of selection dimensions towards an explicit representation of hierarchies, dependencies and constraints. Requirements representations could also be broadened beyond user stories and use cases to accommodate quality, legal, regulatory and normative requirements.

 Finally, the repository should be positioned as an aid to, rather than a substitute for, elicitation. Reuse becomes particularly powerful when combined with a method that first understands the existing situation, identifies the problems to be solved and determines the intended transformation. The resulting combination of As-Is analysis, reusable requirements knowledge, product-line variability and traceable governance requirements could constitute a particularly promising direction for future researches.

 Sources

 [1] Refer 1

 [2] Refer 2

 [3] Refer 3

 [4] Refer 4

 [5] Refer 5

 [6] Refer 6

 [7] Refer 7

 [8] Refer 8

 [9] Refer 9

 [10] Refer 10

 [11] Refer 11

 [12] Refer 12

 [13] Refer 13

 This review was written using GPT-5.6 Sol for translation, text reformulation and english polishing.

Is the work clearly and accurately presented and does it cite the current literature?

Yes

If applicable, is the statistical analysis and its interpretation appropriate?

Not applicable

Are all the source data underlying the results available to ensure full reproducibility?

Partly

Is the study design appropriate and is the work technically sound?

Partly

Are the conclusions drawn adequately supported by the results?

Yes

Are sufficient details of methods and analysis provided to allow replication by others?

Partly

Reviewer Expertise:

Software Engineering, Information Systems, Requirements Engineering, Software Product Lines, Applied Artificial Intelligence

I confirm that I have read this submission and believe that I have an appropriate level of expertise to confirm that it is of an acceptable scientific standard, however I have significant reservations, as outlined above.

## References

[1] Liang, T.-P., & Turban, E. (2011). Introduction to the special issue social commerce: A research framework for social commerce. International Journal of Electronic Commerce, 16(2), 5–14.

[2] Huang, Z., & Benyoucef, M. (2013). From e-commerce to social commerce: A close look at design features. Electronic Commerce Research and Applications, 12(4), 246–259.

[3] Aljassasi, I., & Baghdadi, Y. (2023). A front-end requirement engineering framework of social commerce enterprises (design-phase). In O. K. Hussain, S.-P. Ma, X. Lu, & K.-M. Chao (Eds.), Proceedings of the 2023 IEEE International Conference on e-Business Engineering (ICEBE 2023) (pp. 231–239). IEEE.

[4] Aljassasi, I., Al Hamdani, A., & Baghdadi, Y. (2025). Toward a generic requirement engineering framework for social commerce platforms: Front-end and back-end elements. International Journal of Computing and Digital Systems. University of Bahrain.

[5] Al Jassasi, I., Baghdadi, Y., & Al Hamdani, A. (2025). A generic requirement engineering framework for social commerce platforms: Front-end and back-end features. F1000Research, 14, 1059.

[6] Niu, N., & Easterbrook, S. M. (2008). Extracting and modeling product line functional requirements. In Proceedings of the 16th IEEE International Requirements Engineering Conference (pp. 155–164). IEEE.

[7] She, S., Lotufo, R., Berger, T., Wąsowski, A., & Czarnecki, K. (2011). Reverse engineering feature models. In Proceedings of the 33rd International Conference on Software Engineering (ICSE '11) (pp. 461–470). ACM.

[8] Davril, J.-M., Delfosse, E., Hariri, N., Acher, M., Cleland-Huang, J., & Heymans, P. (2013). Feature model extraction from large collections of informal product descriptions. In Proceedings of the 2013 9th Joint Meeting on Foundations of Software Engineering (pp. 290–300). ACM.

[9] Mefteh, M., Bouassida, N., & Ben-Abdallah, H. (2016). Mining feature models from functional requirements. The Computer Journal, 59(12), 1784–1804.

[10] Dalibor, M., Jansen, N., Rumpe, B., Schmalzing, D., Wachtmeister, L., Wimmer, M., & Wortmann, A. (2022). A cross-domain systematic mapping study on software engineering for Digital Twins. Journal of Systems and Software, 193, 111361.

[11] Glinz, M. (2007). On non-functional requirements. In Proceedings of the 15th IEEE International Requirements Engineering Conference (pp. 21–26). IEEE.

[12] Breaux, T. D., & Antón, A. I. (2008). Analyzing regulatory rules for privacy and security requirements. IEEE Transactions on Software Engineering, 34(1), 5–20.

[13] Yu, E. S. K. (1997). Towards modelling and reasoning support for early-phase requirements engineering. In Proceedings of the Third IEEE International Symposium on Requirements Engineering (pp. 226–235). IEEE.

# Reviewer report: Reviewer response for version 1

**DOI:** 10.5256/f1000research.187548.r510806

**Published:** 2026-08-21

**Authors:** Eric Pardede

**Affiliations:** 1 Latrobe University, Melbourne, Australia

This paper proposes a generic requirement engineering framework for s-commerce platforms, covering both front-end and back-end needs. Built using Design Science Research Methodology, it draws on a systematic literature review to create a reusable knowledge base of requirements, formalised with Feature Description Language and UML. The framework helps developers generate, negotiate and manage platform-specific requirements through variability techniques, and was validated via a prototype tool, expert evaluation and a real case study, giving practical guidance for building more reliable and adaptable s-commerce systems.

 One of the real strengths here is that the paper tackles a gap that's been sitting around for a while, which is most existing s-commerce models are descriptive, they'll tell you what social features should exist but not how to actually engineer them. This framework goes further by covering the whole RE lifecycle, from eliciting and specifying requirements right through to negotiation, validation and ongoing management. It's also genuinely practical rather than just theoretical, since it's backed by a working prototype, a proper knowledge base of front-end and back-end requirements formalised with FDL and UML, and even a recommendation engine that suggests new requirements based on similarity and past usage. The dual front-end/back-end classification is a nice touch too, since it makes sure both the user-facing social features and the less visible system architecture get proper attention. On top of that, the variability management approach means developers aren't stuck with a one-size-fits-all list, they can tailor requirements to their specific platform. The methodology is solid as well, using DSRM plus a mix of prototype testing, meta-criteria comparison against existing frameworks, and expert evaluation from real CIS developers, which gives the findings a decent bit of credibility.

 The main limitation, which the authors themselves admit, is that back-end requirements aren't visualised the way front-end ones are through the prototype, so that side of the framework feels less fleshed out. The evaluation is also fairly conceptual and small scale, with only one case study and a handful of expert participants from a single university, so it's not clear yet how well this holds up across different countries, industries or user behaviours. The reviewer also flagged that most of the references are a bit dated, mainly 2012 to 2020, so the paper doesn't really engage with more recent AI-driven or automated RE approaches. There are a few minor stylistic and spelling slip-ups too, nothing major but worth a tidy up.

Is the work clearly and accurately presented and does it cite the current literature?

Yes

If applicable, is the statistical analysis and its interpretation appropriate?

Not applicable

Are all the source data underlying the results available to ensure full reproducibility?

No source data required

Is the study design appropriate and is the work technically sound?

Yes

Are the conclusions drawn adequately supported by the results?

Yes

Are sufficient details of methods and analysis provided to allow replication by others?

Yes

Reviewer Expertise:

software engineering, databases and data analytics

I confirm that I have read this submission and believe that I have an appropriate level of expertise to confirm that it is of an acceptable scientific standard.

# Reviewer report: Reviewer response for version 1

**DOI:** 10.5256/f1000research.187548.r422565

**Published:** 2025-11-10

**Authors:** Hanny Juwitasary

**Affiliations:** 1 Universiti Teknologi MARA, Puncak Perdana, Malaysia; 2 School of Information Systems, BINUS University, West Jakarta, Jakarta, Indonesia

This article discusses the development of a generic Requirements Engineering (RE) framework for a Social Commerce (s-commerce) platform by integrating front-end (social features and user interaction) and back-end (security, scalability, system architecture) requirements.

 This research is highly relevant to the fields of software engineering and information systems development, particularly in the context of digital transformation and the increasing adoption of social commerce. The article is written systematically and follows the Design Science Research Methodology (DSRM) with clear stages.

 The methods and approaches used in research need to be reconfirmed whether they can be applied in various countries with different user behaviors. This is due to several limitations in this study:

 - The evaluation is still conceptual, with a limited number of participants. A broader field study or comparative experiment with other RE frameworks would strengthen the validity of the findings.

 - The authors acknowledge that visualization of back-end requirements is still lacking. Development of a dashboard or traceability visualization would be a significant enhancement.

 - Although the framework has been tested in an academic setting (Sultan Qaboos University), its applicability to commercial business domains (e.g., retail, e-services) has not been empirically demonstrated.

 - Most references date from 2012–2020; adding more recent literature (2023–2025) on AI-based requirements engineering or automated RE tools would improve relevance.

 - There are minor stylistic and spelling inconsistencies (e.g., in the acknowledgments and table captions) that require minor editing.

Is the work clearly and accurately presented and does it cite the current literature?

Yes

If applicable, is the statistical analysis and its interpretation appropriate?

Partly

Are all the source data underlying the results available to ensure full reproducibility?

Yes

Is the study design appropriate and is the work technically sound?

Yes

Are the conclusions drawn adequately supported by the results?

Yes

Are sufficient details of methods and analysis provided to allow replication by others?

Partly

Reviewer Expertise:

Social commerce, ecommerce, ebusiness, AI, Elearning, Financial Technology

I confirm that I have read this submission and believe that I have an appropriate level of expertise to confirm that it is of an acceptable scientific standard.
